# Regulation of ethylene-responsive *SlWRKY*s involved in color change during tomato fruit ripening

**DOI:** 10.1038/s41598-017-16851-y

**Published:** 2017-11-30

**Authors:** Ling Wang, Xue-lian Zhang, Lu Wang, Yanan Tian, Ning Jia, Shuzhen Chen, Ning-bo Shi, Xuemei Huang, Chu Zhou, Yaowen Yu, Zhao-qi Zhang, Xue-qun Pang

**Affiliations:** 1 State Key Laboratory for Conservation and Utilization of Subtropical Agro-bioresources, South China Agricultural University, Guangzhou, 510642 China; 2 College of Horticulture, South China Agricultural University, Guangzhou, 510642 China; 3 College of Life Sciences, South China Agricultural University, Guangzhou, 510642 China; 4 Guangdong Provincial Key Laboratory of Postharvest Science of Fruits and Vegetables, Guangzhou, 510642 China

## Abstract

WRKY transcription factors (TFs) play important roles in stress responses *in planta*. However, the function of WRKY TFs in the regulation of fruit ripening is unclear. Here, 23 tomato *SlWRKYs* that are similar to ethylene-responsive *WRKY* genes from other plant species, or show up-regulation during fruit ripening in previous genome-wide study, were selected, and their function in fruit ripening was investigated. Twelve *SlWRKYs* were found to be responsive to ethylene (*SlER-WRKYs*), showing expression patterns similar to those of genes related to fruit ripening. Eight *SlER-WRKYs*—SlWRKY16, 17, 22, 25, 31, 33, 53, and 54, detected in the nuclei—interacted with and activated the promoters of 4 genes related to color change: *Pheophytin Pheophorbide Hydrolase* (*SlPPH*), *Pheophorbide a Oxygenase* (*SlPAO*), *Phytoene Synthase 1* (*SlPSY1*) and *Phytoene Desaturase* (*SlPDS*). Yeast two-hybrid and bimolecular fluorescence complement (BiFC) assays in Arabidopsis protoplasts indicated that protein interactions occurred between SlWRKY17 and SlRIN, SlERF2b or SlERF7; SlWRKY33 and SlERF7; SlWRKY54 and SlERF2b; and SlWRKY16 and SlWRKY17. Suppression of *SlWRKY 16*, *17*, *53* or 54 by virus-induced gene silencing (VIGS) retarded the red coloration of the fruit. Our study provides comprehensive molecular evidence that WRKY TFs function in fruit ripening, particularly in color change, and are linked to the intricate regulatory network of other ripening regulators.

## Introduction

The ripening of climacteric fruits is a complex, genetically programmed process that involves dramatic changes in color, texture, flavor, and aroma of the fruits. These changes are initiated by the plant hormone ethylene and coordinated by the expression of a large set of ripening-related genes^1^. The findings in tomato show that ripening is regulated by a number of transcription factors (TFs) in conjunction with ethylene signaling^2^.

Characterization of a number of tomato mutations that display defective ripening has provided novel insights into the control of ripening and has revealed an intricate regulatory network underlying the process^1,3^. Three TFs—the MADS-domain protein Ripening Inhibitor (RIN)^4^, the Squamosa Promoter Binding protein Colorless Non-Ripening (CNR)^5^, and a ripening regulator of the NAC family of TFs, Non-Ripening (NOR)^6,7^—have been proposed to function early in the transcriptional activation cascade upstream of ethylene production^2^. Additional components, including Tomato Agamous-Like1 (TAGL1), Apetala2a (AP2a)^8^, Fruitfull (FUL1 and FUL2), the HD-ZIP protein gene (HB-1)^9^, Ethylene Response Factor6 (ERF6)^10^, and Golden2-Like (GLK)^11^, have also been reported to be associated with early regulators and play important regulatory functions in the fruit ripening process. However, the links between this highly connected regulatory network and downstream effectors that modulate color, texture, and flavor are still poorly understood.

Color change is one of the most obvious traits that accompanies fruit ripening. The ripening stage of tomato fruits can be clearly characterized by the sequential color changing program—green, breaker, turning, orange, light red, red—which is carried out via chlorophyll degradation and lycopene biosynthesis. The chlorophyll (Chl) molecules degrade in a stepwise manner by the action of a series of enzymes, including Chl b reductase (NYC), PPH, PAO and red chlorophyll catabolite reductase (RCCR)^12^. A protein designated SGR (STAY-GREEN) in rice and Arabidopsis has been identified as a positive player upstream of chlorophyll degradation that dismantles Chl-protein complexes^13–16^. The accumulation of lycopene in tomato fruits is correlated with the up-regulation of genes encoding the enzymes functioning in the biosynthesis of the pigment during fruit ripening^17–19^. The expression of two genes encoding the key lycopene biosynthesis enzymes, phytoene synthase (PSY) and phytoene desaturase (PDS), increases rapidly at the breaker stage^20^. Although the biological pathways functioning in color change during fruit ripening have been clearly outlined, knowledge regarding the regulation of the individual genes in the pathways is fragmentary. Most of the information has been obtained when searching for target genes of RIN or other key TFs functioning in early fruit ripening. Using high-throughput chromatin immunoprecipitation with subsequent microarray analysis (Chip-Chip) and transcriptome comparison of the fruit ripening between wild type and *rin* mutants, SGR1 and PSY1 were identified as direct target genes that are positively regulated by RIN^21,22^. In addition to *PSY1* and *SGR1*, other genes related to chlorophyll degradation or lycopene biosynthesis are up-regulated during tomato fruit ripening, but the regulation of these genes is unclear.

WRKY TFs belong to one of the largest plant-specific TF family. By binding to the W-box [(T) TGACC (A/T)] promoter regions of their target genes, WRKY TFs play important biological functions in the modulation of a large set of genes involved in many plant processes^23–25^. WRKY TFs have been found to be involved in the acclimation to various plant stresses, including pathogen infection^26^, drought or cold stress^27,28^. Several stress-related hormone signals triggered by ABA, SA, and JA/MeJA are mediated by WRKY TFs^29–32^. Accumulating evidence also proves the involvement of WRKY TFs in various plant development processes, including seed development^24,33,34^, somatic embryogenesis^35^ and leaf senescence^36^. AtWRKY53 was demonstrated to play an important role in leaf senescence by integrating numerical senescence initiating cues and activating the expression of key senescence-associated genes, such as *SAG12*, *CATALASE 1/2/3* and *ORE9* ^37^. Two *SlWRKYs* (*SlWRKY31* and *SlWRKY23*) were found to increase at both the breaker and red ripe stages of tomato fruit ripening, indicating WRKY TFs may be involved in the regulation of fruit ripening^38^. However, the involvement of WRKY TFs in relation to fruit ripening and color development has not been systematically studied.

In the present study, in order to investigate the regulation of WRKY TFs involved in tomato fruit ripening, 23 *SlWRKY* TFs were selected from the whole gene family, based on their high sequence similarities to 25 ethylene-responsive *WRKY* genes from *Arabidopsis thaliana*, *Oryza sativa*, *Gossypium* (*Gossypium hirsutum* and *Gossypium barbadense*), and *Brassica napus*, or based on their up-regulation profile during fruit ripening in a genome-wide study^39^. Twelve of the *SlWRKYs* were found to be up-regulated by ethylene treatment during fruit ripening and thus were designated *SlER-WRKY*s. They showed overlapping expression patterns with 5 genes related to color change (*SlSGR1*, *SlPPH*, *SlPAO*, *SlPSY1*, and *SlPDS*) and 4 genes related to ripening: 3 genes related to ethylene biosynthesis (*SlACS1*, *SlACO1*, and *SlACO3*) and 1 gene coding polygalacturonase (*SlPG*). W-box elements were found in the promoters of these 9 genes related to fruit ripening. Eight of the SlER-WRKYs were selected to analyze their regulation of the genes related to color change. Furthermore, the interactions between the SlER-WRKYs and SlRIN or SlERFs and interaction among the SlER-WRKYs were analyzed.

## Results

### Phylogenesis analysis of SlWRKYs potentially related to fruit ripening

**Figure 1:**
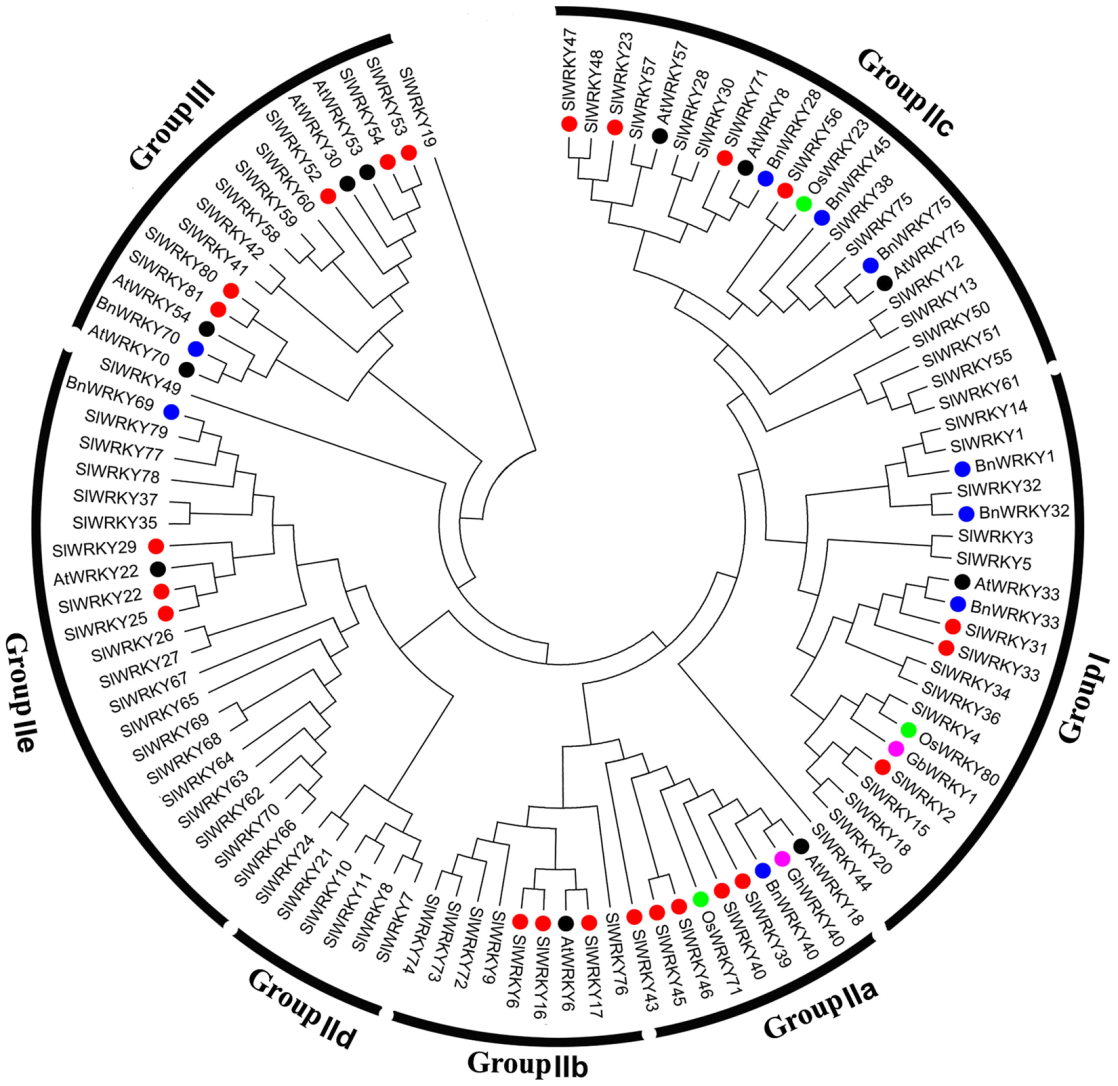
Phylogenetic tree of WRKYs from tomato and other species. Ethylene-responsive or senescence-related WRKYs of *Arabidopsis* (black dot), *Oryza sativa* (green dot), *Brassica napus* (blue dot), and *Gossypium* (*Gossypium hirsutum* and *Gossypium barbadense*, purple dot) were selected based on the literature. The amino acid sequences of 81 WRKYs from tomato (SlWRKYs), 11 from Arabidopsis (AtWRKYs), 3 from rice (OsWRKYs), 9 from *Brassica napus* (BnWRKYs), and 2 from *Gossypium* (GhWRKY40 and GbWRKY1) were aligned using Cluster W2, and a phylogenetic tree was constructed using standard parameters of the neighbor-joining method in MEGA with 1000 bootstrap replicates. Twenty-three SlWRKYs showing high similarity to the 25 WRKYs from the 4 species above were selected for investigation in the present study and labeled with red dots.

All the names of *SlWRKYs* in the present study were issued according to the report of Huang *et al*.^39^ and the Solyc chromosome identifier (https://solgenomics.net)^40^. The relevance of others names as well as the functions proposed in the literature are also listed in Table S1. Phylogenetic analysis was carried out with 81 *SlWRKYs* and 25 senescence-related or ethylene-responsive *WRKY*s from *Arabidopsis thaliana*, *Oryza sativa*, *Gossypium* (*Gossypium hirsutum* and *Gossypium barbadense*), and *Brassica napus* (Supplementary Table S2). Twenty-three *SlWRKY*s of the identified 81 *SIWRKY*s from the tomato genome^39^, which show high similarity to the 25 senescence-related or ethylene-responsive *WRKY*s, or are up-regulated during fruit ripening in a genome wide study^39^, were selected to investigate their involvement in fruit ripening (Fig. 1). The selected *SlWRKYs* were distributed among all subclasses of the *SlWRKY* gene family.

### Expression profiling of genes related to ripening manipulated by ethylene or 1-MCP treatments during fruit ripening

**Figure 2:**
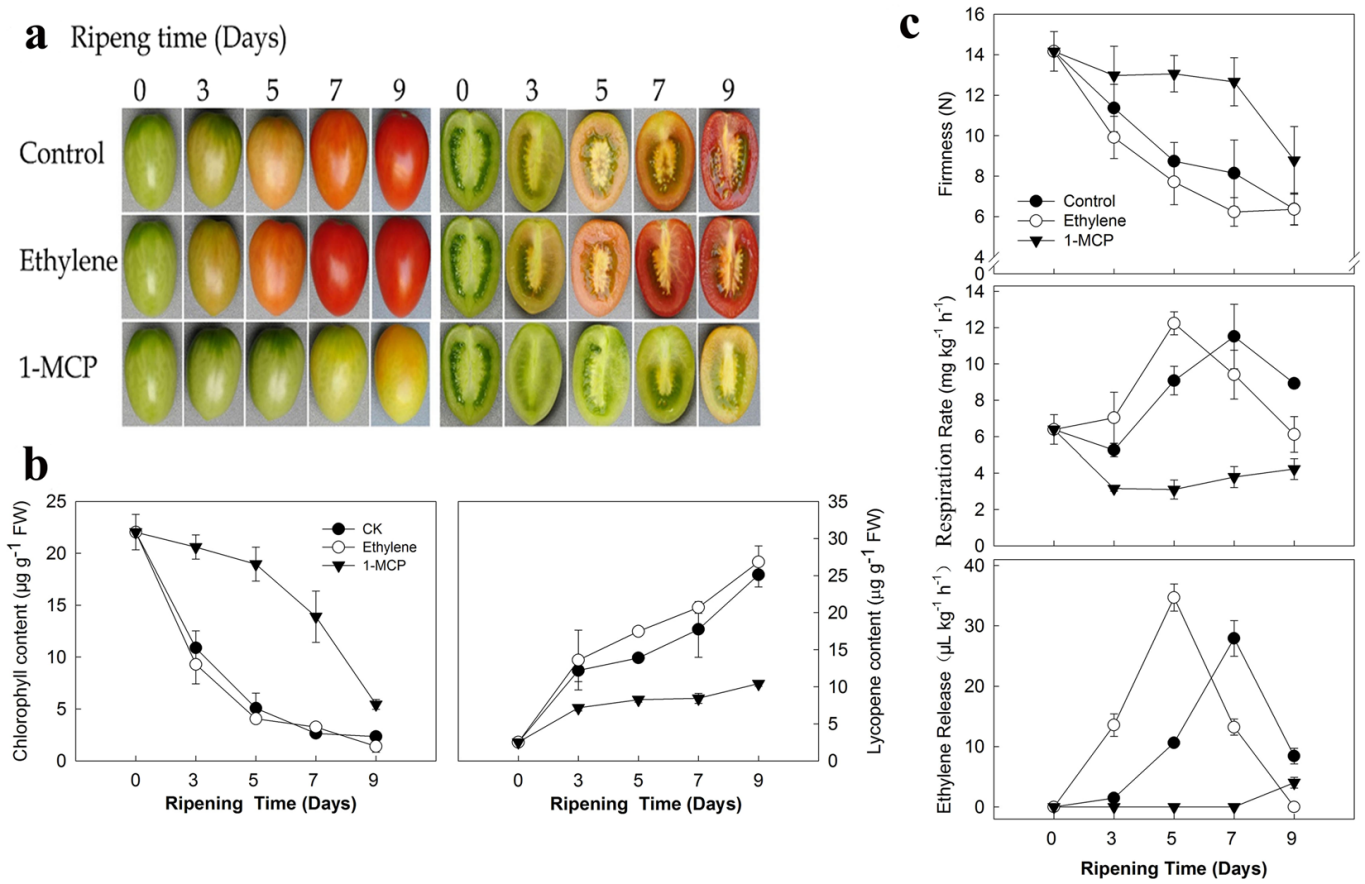
Time course of tomato fruit ripening manipulated by ethylene or 1-MCP. Green mature cherry tomato fruits were treated with 100 µL L^−1^ ethylene or 1 µL L^−1^ 1-MCP for 24 h; no addition of either gas served as the control. All the treated fruit were maintained for another 9 d at 25 °C for ripening. The fruits were removed for imaging purposes and for measuring ripening-related parameters at 3, 5, 7, and 9 d after the treatment. (**a**) Images showed the color change of the fruits. (**b**) Change in total chlorophyll and lycopene contents in the peel tissue during ripening. (**c**) Change in fruit firmness and respiration and ethylene production rates during ripening. Error bars indicate the standard errors (SE) of the values of 3 repeats.

To understand the potential regulation of fruit ripening by the selected *SlWRKY*s, the ripening of the cherry tomatoes was manipulated by ethylene or 1-MCP treatment. Fruits treated with ethylene showed obvious color breaking at 3 d and were fully red at 7 d, which was 2 d earlier than that observed in control fruits. However, color breaking in the 1-MCP-treated fruits was observed only at 9 d (Fig. 2a).

Correlating with color change, lycopene accumulated significantly higher levels in the ethylene treatment than in the control at 5 d and 7 d. The chlorophyll content declined rapidly from 0 to 5 d in the control and ethylene-treated fruits (Fig. 2b). In contrast, the 1-MCP treatment markedly slowed these changes. The decrease in the fruit firmness was accelerated by the ethylene treatment but was significantly delayed by the 1-MCP treatment (Fig. 2c). A respiration rate peak and an ethylene release peak were detected at 5 d for the ethylene-treated fruits and at 7 d for the control fruits. No rate peaks for respiration or ethylene release were detected for the 1-MCP-treated fruits (Fig. 2c). The results showed that fruit ripening was accelerated by the ethylene but slowed by the 1-MCP.

**Figure 3:**
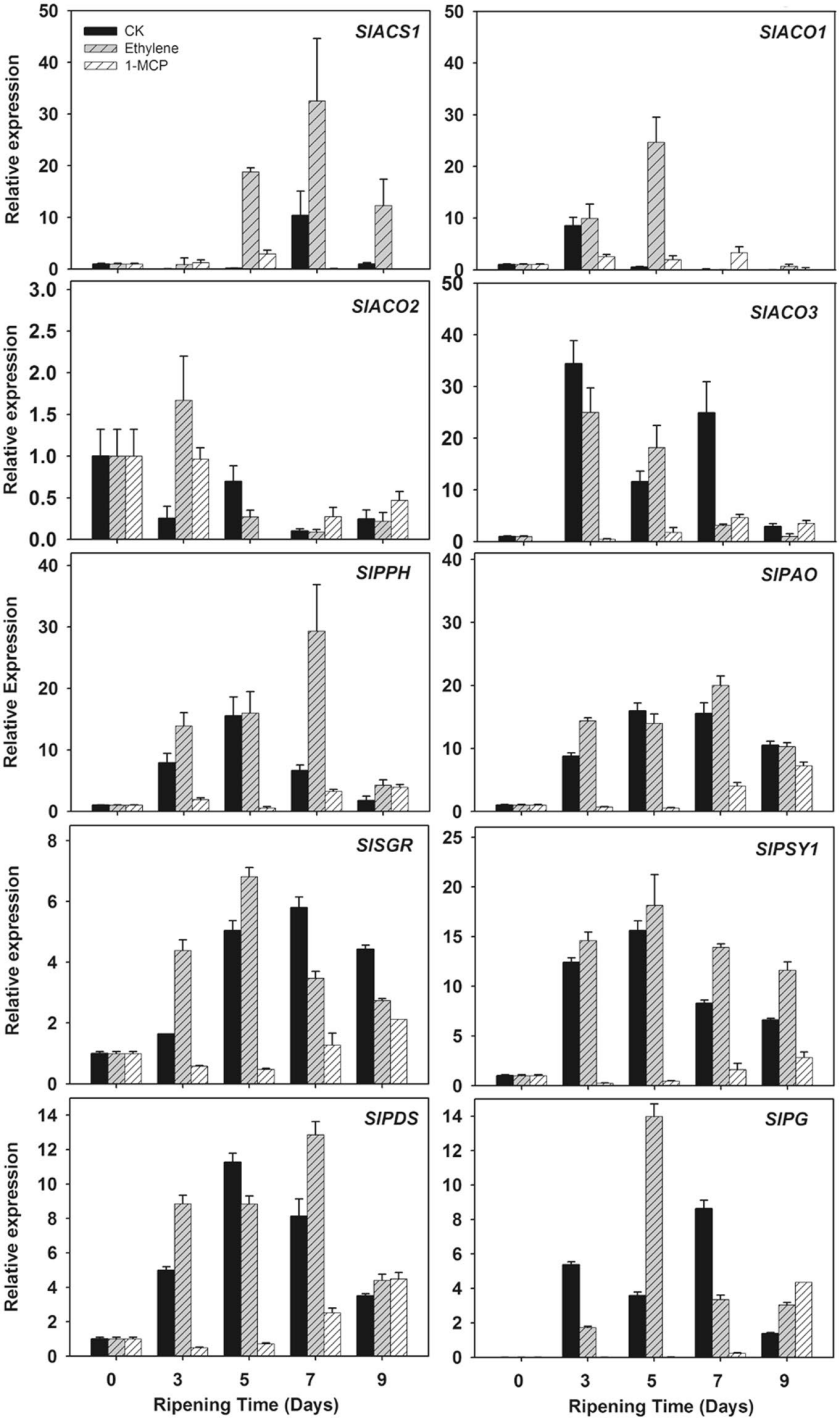
Transcription profiling of the 5 genes related to color change in addition to 4 genes related to ripening. Pericarp tissues of the control and the ethylene- or 1-MCP-treated fruits were sampled at the indicated time points as described in Fig. 2. In the present study, 5 genes were related to color change, 3 were related to ethylene biosynthesis (*ACS1*, *ACO1* and *ACO3*) and one to cell wall metabolism (*PG*). The transcript levels of individual genes in the samples were detected using real-time RT-PCR. Error bars indicate the standard errors (SE) of the values.

The expression patterns of the 4 ethylene biosynthesis genes, *SlACS1*, *SlACO2*, *SlACO1* and *SlACO3*, were clearly induced by ethylene treatment compared to those of the control, while were markedly repressed by 1-MCP (Fig. 3). Three genes related to chlorophyll degradation, *SlPPH*, *SlPAO* and *SlSGR1*, were up-regulated once the ripening process was initiated and peaked at 5 d or 7 d. Similarly, the other two genes related to color change, *SlPSY1* and *SlPDS*, which are key lycopene biosynthesis genes, as well as a gene related to fruit softening (*SlPG*) were highly expressed after ethylene treatment and peaked at 5 d or 7 d. Compared with levels in the fruits of the ethylene treatment and control groups, the expression levels of *SlPPH*, *SlPAO*, *SlSGR*, *SlPSY1*, *SlPDS* and *SlPG* were markedly repressed by 1-MCP treatment throughout the whole ripening developmental process (Fig. 3 and Supplementary Fig. S1b). The expression profiles of these genes related to ripening in response to ethylene and 1-MCP treatments were correlated with the ripening patterns of the fruits described above, confirming their involvement in fruit ripening.

### The expression profiles of 23 selected SlWRKYs

**Figure 4:**
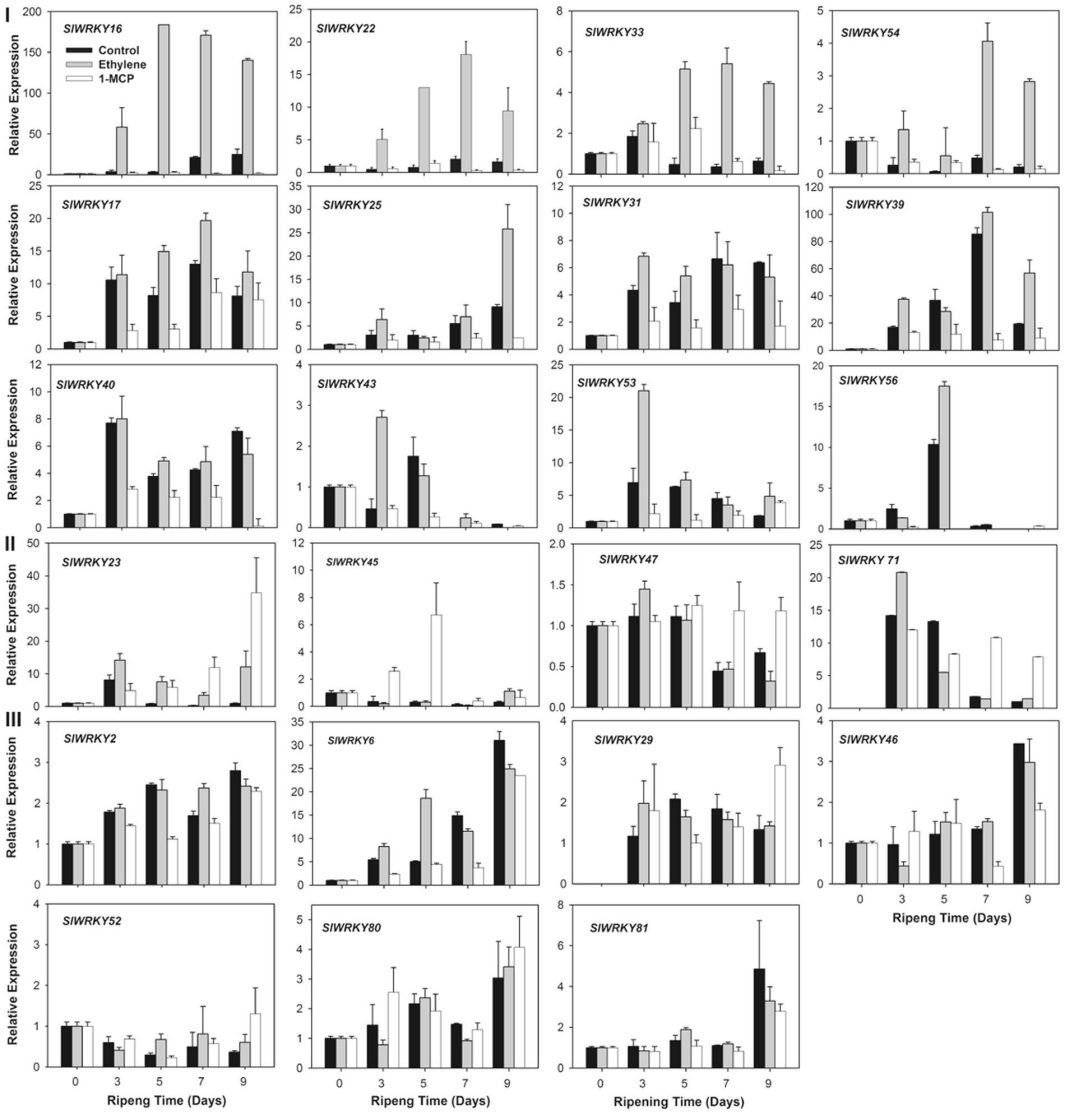
Transcription profiling of 23 selected *SlWRKY* genes during tomato fruit ripening manipulated by exogenous ethylene or 1-MCP. Twenty-three *SlWRKY* genes potentially related to senescence or ethylene were selected from the whole tomato *WRKY* gene family based on their similarities to the relevant genes of other plant species as described in Fig. 1. The transcript levels of individual genes in the samples were detected by real-time RT-PCR. Error bars indicate the standard errors (SE) of the values.

Based on the gene expression profiles of *SlWRKYs* and those related to ripening, a complete linkage hierarchical clustering of these genes was generated. Three different clusters were generated based on the detected expression profiles (Fig. 4 and Supplementary Fig. S1a). In comparison to the fruits of the control and 1-MCP groups, the ethylene treatment apparently induced and maintained high expression levels of the genes throughout the whole ripening process in cluster Ia, including *SlWRKY16*, 22, 33, 40, and *54*. An approximately 150-fold induction by ethylene was detected for *SlWRKY16*. In cluster Ib, *SlWRKY17*, 25, 31 and 39, which were induced by ethylene at the later stage of the ripening, peaked at either 7 d or 9 d. *SlWRKY43*, 53 and 56 in cluster Ic were markedly induced at the early ripening stage by ethylene treatment. The strongest induction was detected for *SlWRKY53*, which was up-regulated approximately 6 and 20 fold in the control and ethylene-treated fruits at 3 d. The *SlWRKY* genes in the cluster I, which showed strong responses to ethylene, were designated *SlER-WRKY*s. The genes of *SlWRKY23*, 45, 47 and 71, which were grouped into cluster II, were up-regulated by 1-MCP, especially in the late ripening period after 7 d, compared to the control. Cluster III contained a large group of genes, including *SlWRKY2*, 6, 29, 46, 52, 80, *and 81*, in which no obvious differences were detected among the control, ethylene and 1-MCP treated fruits.

The search for conserved cis-regulatory elements indicated that the promoter regions of 81 *SlWRKY* genes contained typical ethylene response elements (ERE) and RIN-binding sites (known as CArG boxes, http://bioinformatics.psb.ugent.be/webtools/plantcare/html/). Among the selected 23 *SlWRKY* genes, EREs existed in the promoters of *SlWRKY16*, 17, 22, 23, 45, 47, 52, 54, 71, 80, and 81, and CArG boxes existed in the promoters of *SlWRKY6*, 17, 22, 25, 29, 31, 33, 39, 45, 46, 47, 52, 53, 54, 80, and 81 (Supplementary Fig. S2). Interestingly, 9 of the 12 *SlER-WRKY* genes in cluster I, *SlWRKY16*, 17, 22, 25, 31, 33, 39, 53 and 54, contained EREs, CArG boxes or both elements in their promoter regions, which correlated with the strong responses to ethylene (Fig. 4 and Supplementary Fig. S1a). These genes may function in the regulation of tomato fruit ripening through a direct or indirect ethylene response. Thus, among the 12 *SlER-WRKY*s, 8 genes, namely *SlWRKY16*, 17, 22, 25, 31, 33, 53 and 54 (Supplementary Fig. S1a, highlighted by red), had higher expression than those in the same cluster and were therefore selected for further investigation of their function in color change related to fruit ripening. Notably, ERE element was also detected in *SlWRKY 16*, *17*, *22*, *and 54*, whose responses to ethylene in fruit are required to be confirmed. More SlWRKYs that are potentially involved in fruit ripening may be identified when detailed transcriptome analysis is applied.

The expression patterns of the 8 selected *SlER-WRKY* genes were also analyzed during leaf development, natural fruit ripening in plants, and *rin* tomato mutations that displayed defective ripening. Strong up-regulation was detected for all 8 genes in early-senescence (ES) leaves, with 200-fold higher transcript levels of *SlWRKY25*, 33 and 54 than those observed in young leaves (Supplementary Fig. S3). The up-regulation of the 8 *SlER-WRKY* genes, which was observed in fruit ripening after harvest, was also observed during natural fruit ripening in the plants, with strong induction at the pink and red ripe stages (Supplementary Fig. S4). These results further indicated that the 8 *SlER-WRKY* genes may be senescence genes related to ripening. Interestingly, in comparison to the WT, the expression of the 8 *SlER-WRKY* genes were not repressed in *rin*, instead by 2–8 folds up-regulated, unlike the expression of 4 typical fruit ripening related genes, *SlPSY1*, *SlPG*, *SlACS2*, and *E4*, which were dramatically depressed in *rin* (Supplementary Fig. S5).

### The *SlER-WRKYs* are localized to the nucleus and function as potential transcriptional activators

**Figure 5:**
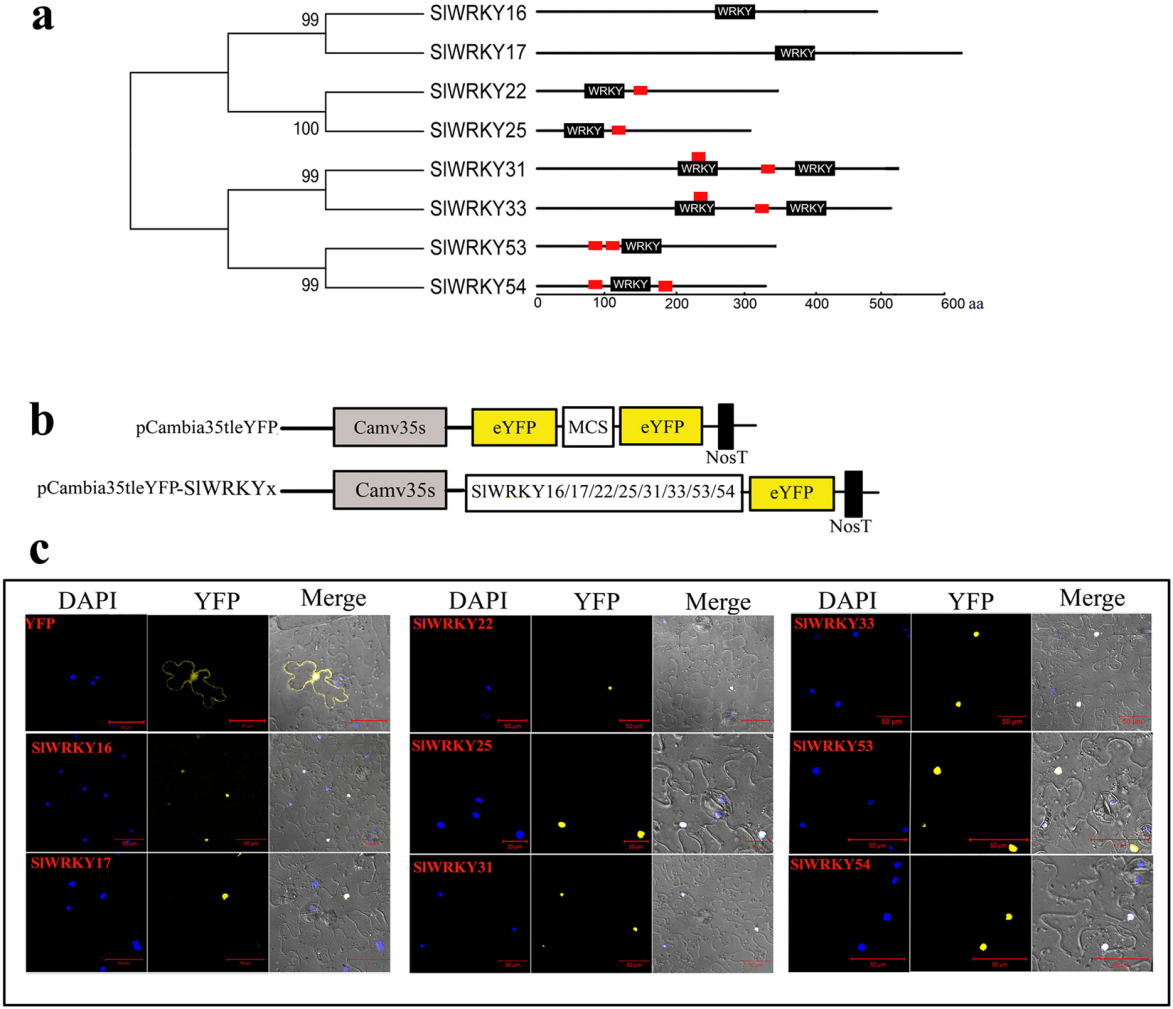
Subcellular localization of the 8 SlER-WRKYs and their transcriptional activities in yeast. (**a**) A neighbor-joining phylogenetic tree of the 8 ethylene-responsive SlWRKYs was created using MEGA 4.1 software, showing WRKY domains and the putative nuclear localization signals (NLS) (red box) predicted by WoLFPSORT program (http://wolfpsort.org/). (**b**) The schematics of the empty vector (35S::YFP) and the 35S::YFP-SlER-WRKY vectors show the expression of *YFP* only and the in-frame expression of the 8 *SlER-WRKY* ORFs with *YFP*, respectively. (**c**) Transient expression of the 35S::YFP and 35S::*YFP-SlER-WRKY* constructs in tobacco epidermal cells. Yellow fluorescence corresponding to the expressed proteins was observed with a confocal microscope 24 h after transient transformation mediated by *Agrobacterium*. The nuclei of the tobacco cells were visualized by DAPI staining. Images were taken in a dark field for yellow fluorescence, while the outline of the cell and the bright-field images were merged. The bars shown in the images are 50 μm.

**Figure 6:**
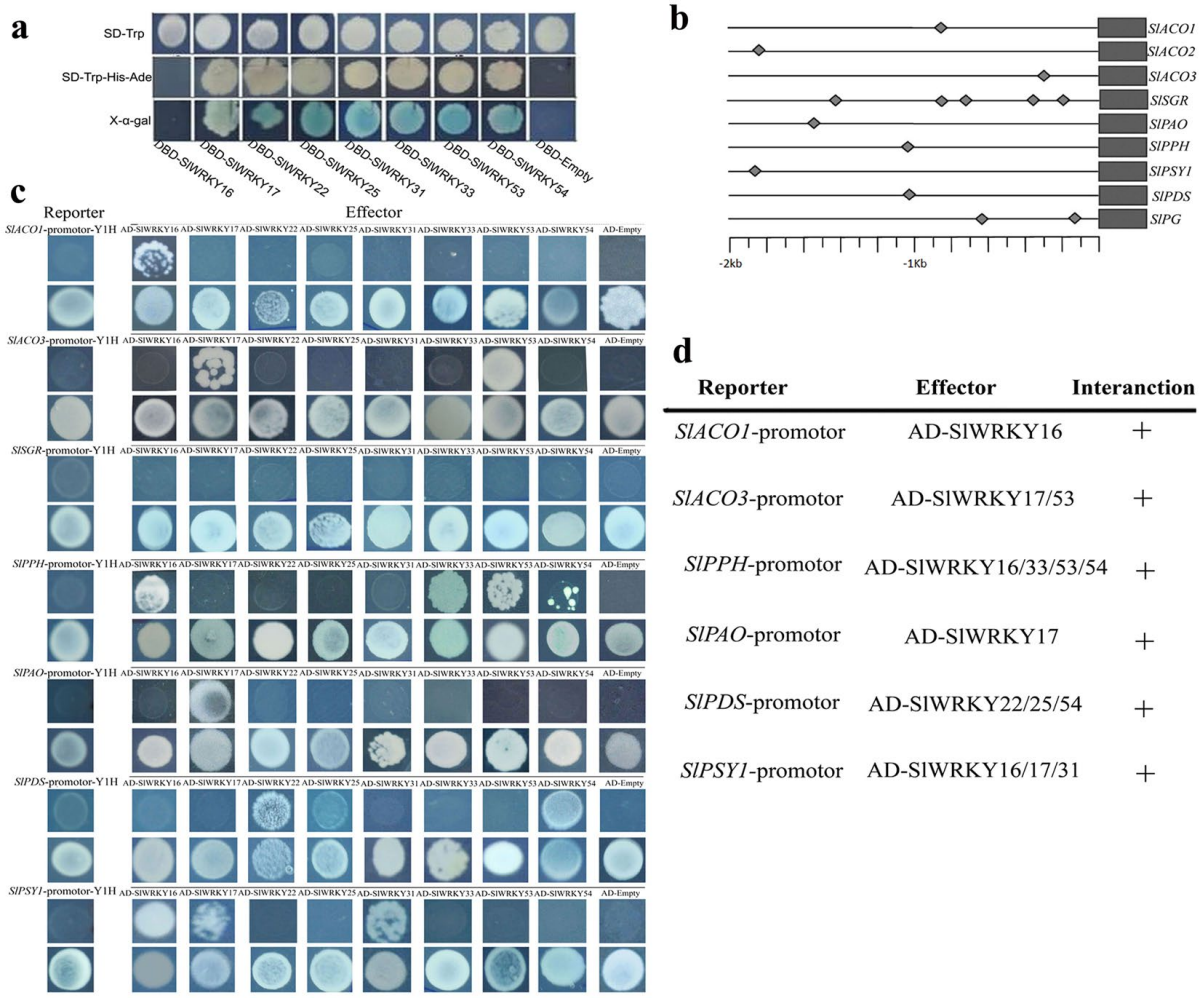
Transcriptional activities of the SlER-WRKYs and their interaction with the promoters of the genes related to color change. (**a**) Transactivation of the *SlER-WRKY* genes in yeast. Y2HGold yeast cells transformed with the pGBKT7 harboring the 8 *SlER-WRKY* ORFs were grown on SD/-Trp or SD/-Trp-His-Ade media, and the α-galactosidase activity was determined. An empty pGBKT7-BD vector was used as a control. Three independent experiments were performed. (**b**) Schematic diagram of the promoters of the relevant genes are indicated with a line (promoter length) and diamonds (W-box elements). (**c**) Yeast one-hybrid analysis of the interaction of the SlER-WRKYs with the promoters of *SlPAO*, *SlPPH*, *SlSGR*, *SlPSY1*, *SlPDS*, *SlACO1*, and *SlACO3*. No basal activation of the promoters was observed for the yeast strains harboring the promoter-Y1H reporter grown on SD/–Ura medium with ABA. Yeast growth assays after the promoter-Y1H reporter strains were transformed with plasmids carrying cassettes constitutively expressing *SlWRKY*s (effector). Interaction was determined based on the ability of the transformed yeast strains to grow on SD medium lacking Leu in the presence of 50–300 ng mL^−1^ ABA. (**d**) The interaction of the SlER-WRKYs with the promoters of *SlPAO*, *SlPPH*, *SlSGR*, *SlPSY1*, *SlPDS*, *SlACO1*, and *SlACO3* detected in the yeast one-hybrid analysis (C) was summarized.

The sequences of the predicted SlER-WRKY proteins were analyzed using WoLFPSORT (http://wolfpsort.org/), which indicated that 6 of the SlER-WRKYs (SlWRKY16 and 17 were excluded) contain 1 to 2 putative nuclear localization signals in addition to the WRKY domains (Fig. 5a). To confirm their nuclear localization, individual SlER-WRKYs fused to yellow fluorescent protein (YFP) (Fig. 5b) were transiently expressed in tobacco epidermal cells under the control of CaMV35S promoter. All 8 SlER-WRKYs exclusively co-located with nuclear DAPI dye, indicating their nuclear location in the cells (Fig. 5c). Transcriptional activity assays of the 8 SlER-WRKY proteins showed that, except for *SlWRKY16*, Y2HGold yeast cells transformed with pGBKT7 harboring the 7 *SlER-WRKY* ORFs grew on selective media without SD/-Trp-His-Ade and showed α-galactosidase activities, indicating that SlWRKY17, 22, 25, 31, 33, 53, 54 proteins have transcriptional activity in yeast (Fig. 6a).

### Interactions of the SlER-WRKYs with the promoters of genes related to color change

As shown in Fig. 6b, at least one putative W-box element in the 2-kb promoter sequences upstream of the *SlACO1*, *SlACO3*, *SlPAO*, *SlPPH*, *SlSGR1*, *SlPSY1* and *SlPDS* genes and 5 W-box elements were detected in the promoter of *SlSGR1*.

The interaction of the SlER-WRKYs with the promoters of the genes related to color change were analyzed using an Y1HGold System (Clontech, USA). After transforming pGADT7-*SlWRKY16* plasmids to constitutively express *SlWRKY16*, Y1HGold reporter cells harboring the *SlACO1* promoter grew in the presence of ABA (100 ng ml^−1^), indicating an interaction between SlWRKY16 and the promoter of *SlACO1*. Similarly, the growth of the Y1HGold cells after being transformed with the plasmids expressing the other *SlER-WRKYs* in the presence of ABA indicated that interaction was also observed for SlWRKY17 or 53 and the *SlACO3* promoter (*pSlACO3*); for SlWRKY16, 33, 53 or 54 and *pSlPPH*; for SlWRKY17 and *pSlPAO*; for SlWRKY16, 17 or 31 and *pSlPSY1*; and for SlWRKY22, 25 or 54 and *pSlPDS*. No interaction was observed for any of the SlER-WRKY*s* and *pSlSGR1* (Fig. 6c and d).

**Figure 7:**
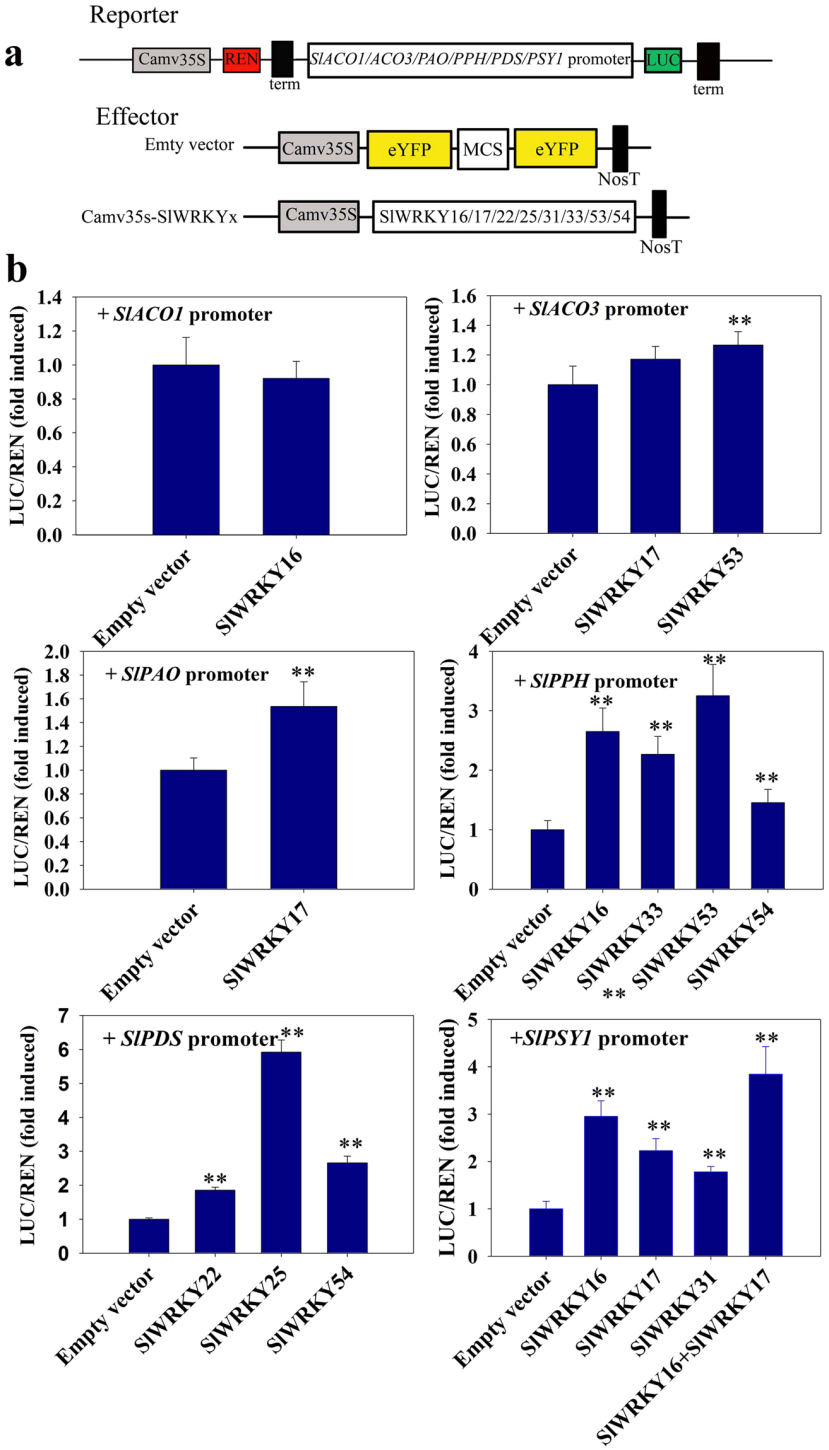
Activation of the gene promoters related to color change by the SlER-WRKYs. (A) The schematics of the reporter and effector vectors. For the reporter, the expression of the *LUC* and *REN* genes was driven by the promoters of the genes related to ripening and by p35S, respectively. The expression of the S*lER-WRKY*s promoted by p35S served as the effectors. (**a**) Dual luciferase analysis of the activities of the promoters by the SlER-WRKYs. The analysis was carried out only for those showing interaction in the Y1H analysis (Fig. 5b). Error bars indicate the standard errors (SE) of the values of 6 repeats. **P < 0.01 indicates significant differences (t-test) compared with the empty vector.

The above interaction of the SlER-WRKYs and the promoters of the 4 color change- and 2 ethylene-related genes was further confirmed using an *in vivo* dual luciferase assay via a tobacco transient co-transformation system (Fig. 7a). Significant activation was observed for WRKY16 or 53 on *pSlPPH*; SlWRKY17 on *pSlPaO*; SlWRKY16, 17, 31, or 33 on *pSlPSY1*; and SlWRKY22, 25 or 54 on *pSlPDS* (Fig. 7b). Strong activation was detected for SlWRKY53 on *pSlPPH*, SlWRKY25 on *pSlPDS*, and SlWRKY16 on *pSlPSY1*, with 3.3-, 6- and 2.9-fold inductions, respectively. No significant induction was observed for the tested SlER-WRKYs on *pSlACO1* or *pSlACO3*.

Based on the results from the Y1H and *in vivo* regulation assays, SlWRKY16, 17, 22, 25, 31, 33, 53, and 54 may bind and activate the promoters of genes related to color change, suggesting that these SlWRKYs may be involved in the regulation of color change during tomato fruit ripening.

### Interaction of the SlER-WRKYs and other key ripening-related TFs

Based on the above results, SlWRKY16 and 17 could interact with and activate the promoters of 3 color-change genes (Figs 6 and 7), and their expression was strongly induced by ethylene treatment during fruit ripening (Fig. 4 and Supplementary Fig. S1). Accordingly, the putative interactions of SlWRKY16 or 17 with the other 6 SlER-WRKYs, as well as the interaction of SlMADS-RIN^22,41^, SlERF7^42^ or SlERF2b^43^ with the 8 SlER-WRKYs were tested using Y2H assays and BiFC.

**Figure 8:**
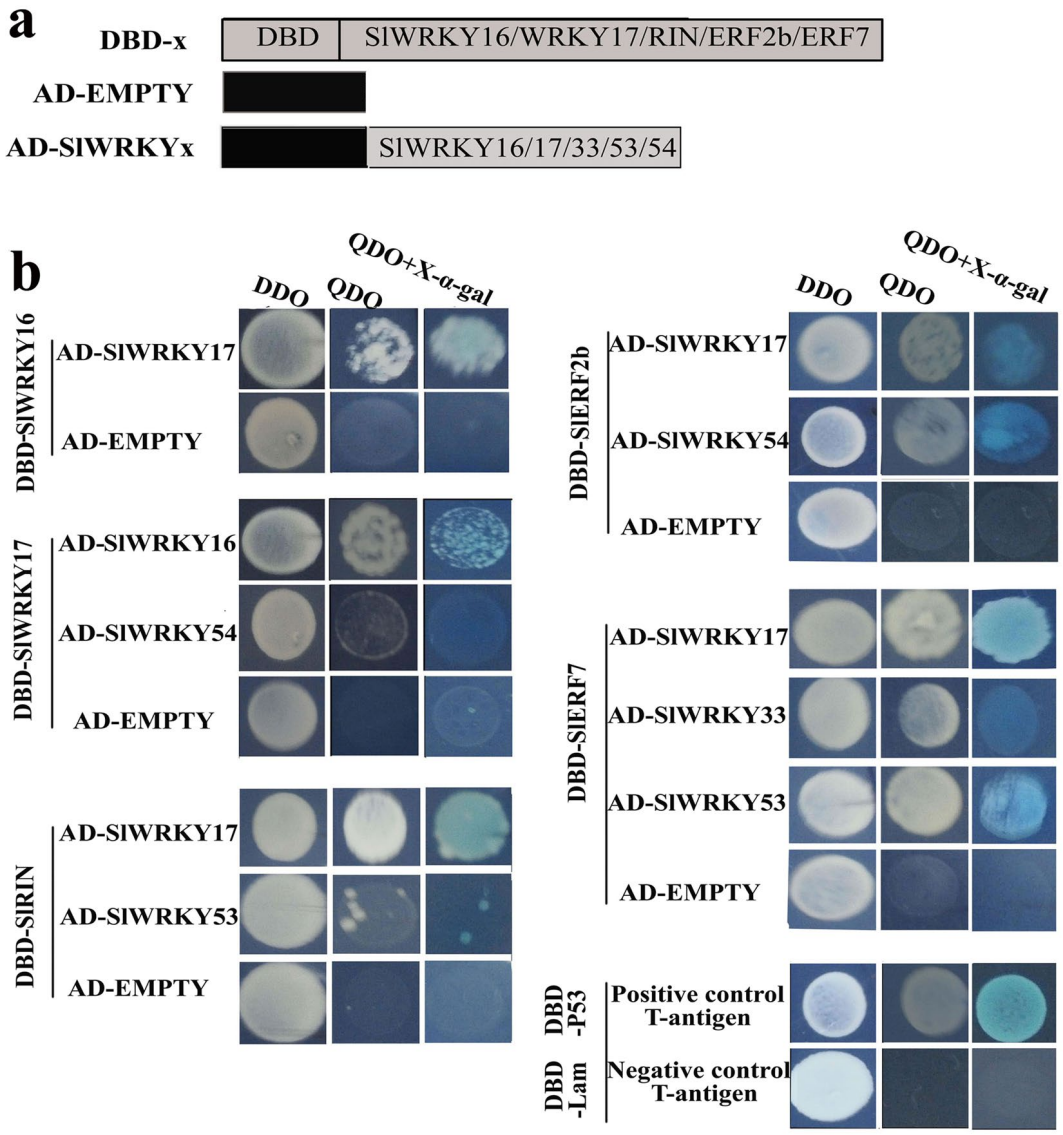
Yeast two-hybrid analysis for interactions between the SlER-WRKYs and other key ripening-related transcription factors. (**a**) The coding regions of *SlWRKY16/17*, *SlRIN*, and *SlERF2b/7* were cloned into the pGBKT7 vector to create the DBD-constructs. The coding regions of the *SlER-WRKY*s were cloned into the pGADT7 vector to create the AD-constructs. (**b**) Yeast two-hybrid analysis for the interaction between SlWRKY16 and 17 and for the interactions between SlRIN, SlERF2b or SlERF7 and SlWRKY17, 33, 53 or 54. The Y2H strain harboring the indicated plasmid combinations was grown on either the SD/-Leu/-Trp nonselective media (DDO), SD/-Leu/Trp/-His/-Ade/AbA selective media (QDO) or QDO followed by X-gal staining (QDO + X-α-gal). Control tests for each assay were the transformants of the pGBKT7-SlWRKY16/SlWRKY17/SlRIN/SlERF2B/SlERF7 with empty pGADT7 vectors (A). The transformants with pGBKT7–53 and pGADT7-T as well as pGBKT7-Lam and pGADT7-T served as positive and negative controls, respectively.

As shown in Fig. 8b, yeast cells co-transformed with a positive control pair (pGBKT7−53 + pGADT7-T) with the pairs of DBD-SlWRKY17 and AD-SlWRKY16, DBD-SlWRKY16 and AD-SlWRKY17, or DBD-SlWRKY17 and AD-SlWRKY54 grew well on QDO selective media and showed α-galactosidase activity on QDO/X/A indicator plates, indicating interactions between SlWRKY17 and SlWRKY16, SlWRKY17 and SlWRKY54. The interaction between SlWRKY17 and SlWRKY16 was more prominent than the interaction of SlWRKY17 and SlWRKY54. No interactions of SlWRKY16 or SlWRKY17 with other SlER-WRKY TFs were observed (data not shown).

Similar cell growth and α-galactosidase activity were detected for yeasts transformed with a random combination of DBD-SlRIN/SlERF2b/SlERF7 and one of the 8 AD-SlER-WRKYs (Fig. 8a). The results suggested that interaction may occur between SlRIN and SlWRKY17; SlERF2b and SlWRKY17 or SlWRKY54; and SlERF7 and SlWRKY17, SlWRKY33, or SlWRKY53 (Fig. 8b). The other combinations of SlRIN, SlERF2b or SlERF7 and the other SlER-WRKYs did not grow on QDO plates (data not shown), indicating no interactions.

**Figure 9:**
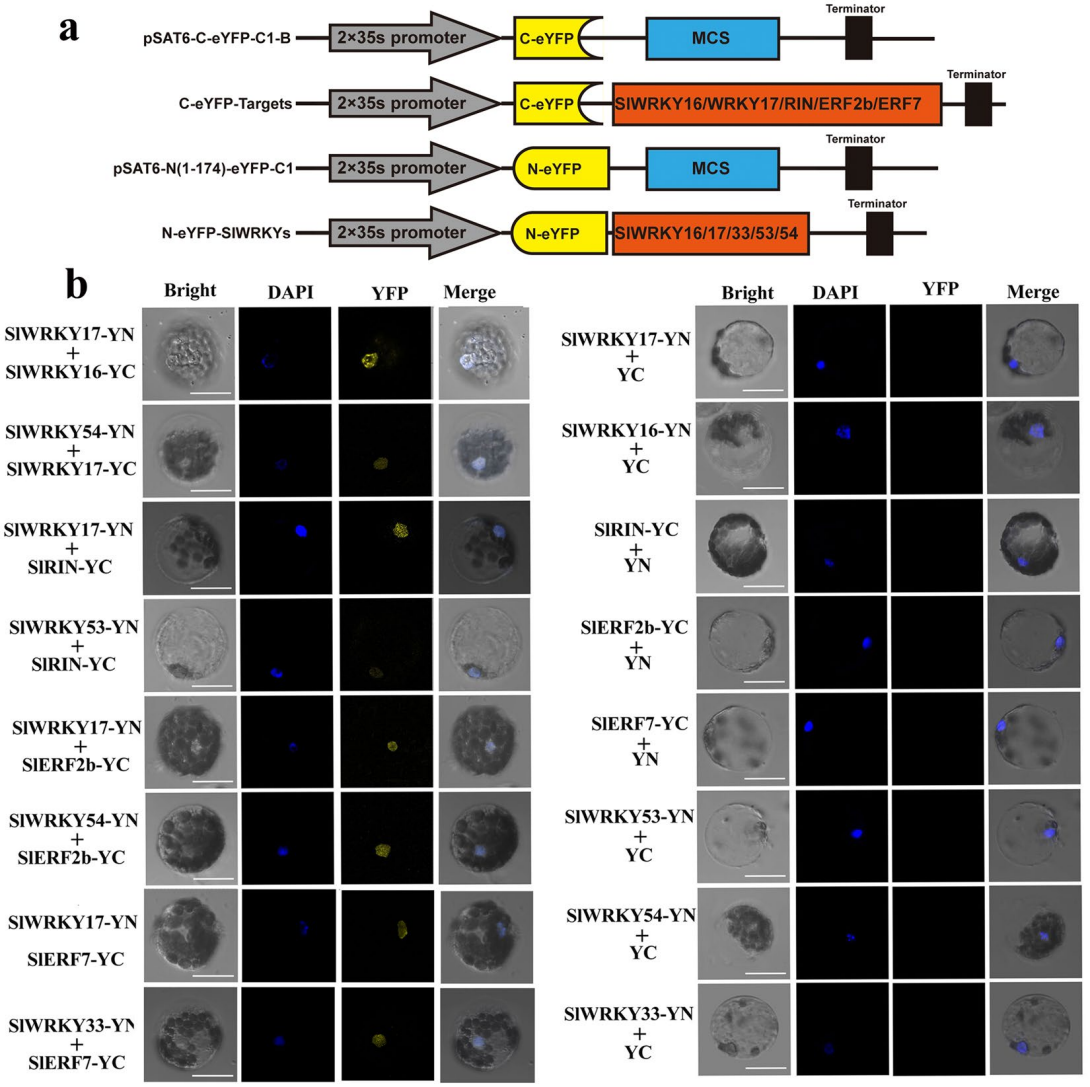
BiFC visualization of the interactions of SlER-WRKYs and other ripening-related transcription factors in transiently co-expressed Arabidopsis protoplasts. (**a**) ORFs of *SlWRKY 16/17*, *SlRIN*, *SlERF2b* and *SlERF7* were expressed in frame with the C (YC)-terminus of *YFP*. In addition, *SlWRKY*17, 33, 53 and 54 were expressed in frame with the N (YN)-terminus of *YFP*. The expression of the N (YN)- or C (YC)-termini of YFP alone was used for a control. (**b**) The pairs of the YN-fused constructs and the YC-fused constructs were transiently co-expressed in Arabidopsis protoplasts. Co-expression of YN-fused and YC-only constructs or YC-fused with YN-only constructs served as the controls. Yellow fluorescence generated by the interaction of the relevant proteins was observed with a confocal microscope 16 h after the transient transformation of the plasmids mediated by PEG. Imaging of the YFP and the nuclei staining were as described in Fig. 5c. The bars shown in the images are 20 μm.

BiFC assays were performed to further confirm the interaction results obtained by the Y2H assay. Robust YFP fluorescent signals, which co-located with DAPI nuclear dye, were detected in Arabidopsis protoplasts when co-expressed with SlWRKY17-nEYFP and SlWRKY16-cEYFP, SlWRKY17-nEYFP and SlRIN-cEYFP, SlWRKY17/SlWRKY54-nEYFP and SlERF2b-cEYFP, and SlWRKY17/SlWRKY33-nEYFP and SlERF7-cEYFP (Fig. 9a), which correlate with the interactions detected for these pairs of TFs in Y2H assays (Fig. 8). The weak florescent signals were observed in protoplasts when co-expressed with WRKY54-nEYFP and SlWRKY17-cEYFP, SlWRKY53-nEYFP and SlRIN-cEYFP, which correlated with the weak interactions of these proteins detected in the Y2H (Fig. 9b). No YFP signal was observed in the protoplasts that expressed only one of the components of the above pairs of TFs (Fig. 9b). According to the Y2H and BiFC assays, interactions between the ethylene-responsive SlER-WRKYs and the key ripening-related TFs may occur.

### Virus-induced gene silencing (VIGS) detecting *SlWRKY* expression in tomato fruit

**Figure 10:**
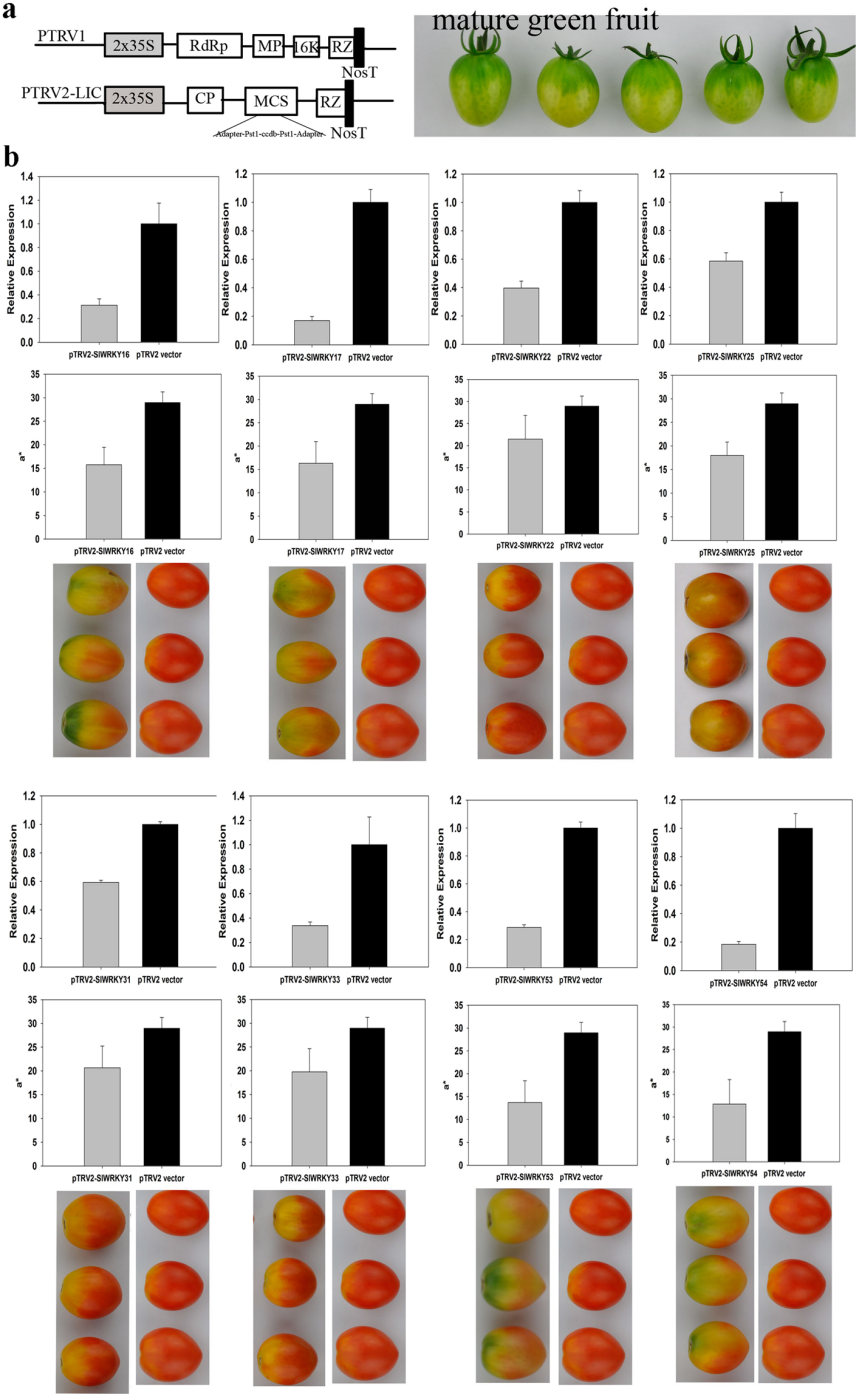
VIGS based transient gene silencing of the 8 *SlER-WRKY* genes during tomato fruit ripening. (**a**) cDNA fragments of *SlER-WRKY 16*, *17*, *22*, *25*, *31*, *33*, *53 and 54* were inserted into the MSC sites in pTRV2-LIC vectors respectively and the Agrobacteria GV1303 harboring the pTRV2-LIC-SlER-WRKYx and pTRV1 were infiltrated in the green mature tomato fruit in the plants at 30 DPA. Infiltration of the bacteria harboring pTRV2-LIC and pTRV1 served as empty vector control. (**b**) The relative expression of the 8 SlER-WRKY genes, the red color a* values and the appearance of the tomato fruits at 14 days after infiltration were shown in a same column. Infiltration for invidious gene was repeated 3 times with 10 fruit for one repeat. Gene expression was analyzed for each fruit and the fruit with efficient silencing were subjected for a* value measurement. The data obtained from 6 representative fruit for each gene were used for statistics analysis. The gene expression level in control fruits was standardized to unity in each case. Bars indicate standard errors of the means.

To further explore the function of the 8 SlER-WRKY TFs in the color change during tomato ripening, VIGS were performed at 30 DPA (Fig. 10a). The efficiency of the silencing was firstly confirmed by evaluation of silencing an ethylene bio-synthesis gene, *SlACS4* ^42^. Compared to the empty vector control, the expression of *SlACS4* was reduced by 58% at 14 days after infiltration, and un-even ripening was observed (Fig. S8). As efficiently silencing of *SlACS4*, the transcription of *SlER-WRKY 16*, *17*, *22*, *25*, *31*, *33*, *53 and 54* were down regulated by 69, 83, 60, 41, 41, 66, 71 and 82% respectively at 14 days after infiltration. Obviously uneven coloration was observed for the silencing of *SlER-WRKY 16*, *17*, *53 and 54*, with 45, 44, 53, 56% reduction in a* values when compared to the fruit of empty vector controls. The reduction of a* values was slight for the silencing of *SlER-WRKY 22*, *25*, *31 and 33* (Fig. 10b). The results further indicate that *SlER-WRKY 16*, *17*, *53 and 54* may participate in the color control during tomato fruit ripening.

## Discussion

Fruit ripening is a complex developmental process that is coordinated with the up-regulation of a large set of ripening-related genes, which are regulated by many critical TFs, including RIN, CNR, and NOR^4,5,44^. WRKYs are plant-specific TFs mainly involved in stress resistance responses^26,45–47^ as well as in various developmental processes, including leaf senescence^48–51^. However, whether and how the SlWRKY TFs regulate the ripening process of tomato fruit still remain uncertain. In the present study, 23 *SlWRKY*s that showed high similarity to the senescence- or ethylene signaling-related *WRKY*s from other species, or showed up-regulation during fruit ripening, were selected from the 81 total *SlWRKY*s in the tomato genome^39^. Among the 23 selected genes, 12 *SlWRKY* were dramatically up-regulated by ethylene but were repressed by 1-MCP during tomato fruit ripening, designated as *SlER-WRKY*s. Eight of the SlER-WRKYs were found to potentially regulate 4 genes related to color change and to interact with other key ripening-related TFs, which indicates that these members may be involved in the regulation of color change during tomato fruit ripening.

WRKYs are mainly involved in responses to stress and in the process of leaf senescence^24^; thus, many *WRKY* genes were also found to be regulated by ethylene. In *Brassica napus*, *BnWRKY1*, 28, 32, 33, 40, 45, *69 and 75* were clearly induced by ethylene treatment, and most of these genes also responded to fungal pathogens^52^. The expression level of *OsWRKY23* increased during continuous dark-induced leaf senescence, and the level also increased rapidly within 1–4 h after rice seedlings were treated with ACC^53^. In the present study, 12 ethylene-responsive *SlER-WRKY* genes, *SlWRKY16*, 17, 22, 25, 31, 33, 39, 40, 43, 53, 54 and 56, were identified based on the up-regulation of genes in ethylene-treated fruit compared to that of the control and 1-MCP-treated fruit. Bartley and Ishida^38^ found that the expression of *SlWRKY31* and *SlWRKY23* increased at the breaker and red ripe stages of tomato fruit ripening in plants. *SlWRKY24* and *SlWRKY37* were detected to be putatively involved in the regulation of tomato fruit ripening^54^. RNA-Seq analysis of 81 *SlWRKY*s in various cultivars or subspecies of *Lycopersicum* fruits showed higher expression levels of *SlWRKY16*, 17, 22, 25, 31, 39, 40, 43, *and 53* at the breaker stage than at the mature green stage (Supplementary Fig. S6)^55^. The presence of some highly expressed genes in a previous RNA-Seq analysis that were also selected in the present study but not identified as *SlER-WRKY*s, such as SlWRKY 2, 6, 8, and 1, may be due to either the similarity of their expression patterns to those of the control, or to induction from 1-MCP. Furthermore, the 8 *SlER-WRKY*s were not repressed in *rin*, a ripening mutant of tomato, instead they were 2–8 folds up-regulated after harvest when compared to the WT. We estimate that, the MADS-RIN may be an important factor for fruit ripening, to compensate for the lesion of the gene, the mutant fruit activated the WRKYs to by-pass the RIN-pathway to enforce limited ripening for seed dispersal^3^, indicating the WRKYs were required for the network of the fruit ripening.

Plant WRKY TFs regulate gene expression by binding to the W-box elements in promoter regions of their target genes^23,56^. A number of candidate target genes of AtWRKY53 were isolated by genomic pull-down assays using recombinant AtWRKY53 protein. The promoter sequences of most of the target genes contained one or more W-boxes, and the interaction of AtWRKY53 with these different promoters has been confirmed by EMSA (*in vitro*) and *in vivo* regulation assays^51^. Based on bioinformatics analyses, W-box elements were found in the promoter sequences of genes that involved in ethylene, cell wall, chlorophyll, carotenoid metabolisms, as well as several ripening regulatory TFs for tomato fruit (Supplementary Fig. S7). The yeast one-hybrid analysis in the present study showed that 8 selected ethylene-responsive SlER-WRKYs interacted with 4 genes related to color change, *SlPAO*, *SlPPH*, *SlPSY1* and *SlPDS*, and 2 genes related to ethylene biosynthesis, *SlACO1* and *SlACO3*, showing the preferential binding of the SlER-WRKYs (Fig. 6b and c). A similar phenomenon has been observed in that AtWRKY18, AtWRKY60 and AtWRKY4 could interact with the W-box in the promoters of *AtABI4* and *AtABI5* genes, whereas the 3 WRKYs have their own preferential binding domains in the two promoters^57^. None of the tested SlWRKYs could bind to the promoter of *SlSGR1* in our study, even though the promoter was detected to contain 5 W-box elements. This could be due to W-box-specific binding or the distinct arrangement of functional sequences, resulting in select WRKY factors with defined genes, thereby leading to distinct transcriptional outputs^23^.

Chlorophyll degradation is considered the initial step in color change during fruit ripening^58^. However, the regulation of genes related to chlorophyll degradation in fruit ripening is not well known. By the comparison between the fruit ripening of wild type tomato and *rin* mutants, *SlSGR1*, a gene functioning upstream of chlorophyll degradation, was identified as the direct target gene positively regulated by RIN^22^. In the present study, SlER-WRKY16, 17 and 53 were found to directly interact with the promoters of *SlPAO* and *SlPPH*, functioning downstream of SGR and are up-regulated during leaf senescence^12,59^ and quickly activated after ethylene treatment during banana and pear fruit ripening^58,60^. Lycopene accumulation is also a characteristic process of tomato fruit ripening, and several genes functioning in the carotenoid biosynthesis pathway have been identified as the direct targets of RIN. These genes include 4-diphosphocytidyl-2-C-methyl-D-erythritol kinase (ISPE), z-carotene isomerase (Z-ISO) and carotenoid isomerase (CRTISO)^22^. In the present study, interaction and strong activation of the *SlPDS* gene were detected for SlWRKY25 and 54. One of key synthesis genes, phytoene synthase (*PSY1*), was found to be a direct target gene of both RIN^44,61^ and FUL1^62^. Recently, it was reported that RIN, FUL homologs, and tomato AGAMOUS-LIKE1 may form DNA-binding complexes, co-regulating fruit ripening^41^. In the present study, the interaction and activation of the *SlPSY1* gene were detected for SlWRKY16, 17, and 31 (Figs 6 and 7). Interestingly, the co-overexpression of SlWRKY16 and 17 in tobacco leaves resulted in stronger activation of the *SlPSY1* promoter than that from either of the two individually (Fig. 7), and these two TFs were observed to interact with each other (Figs 8b and 9b) based on the results of the yeast two-hybrid and BiFC assays.

The interaction of the SlER-WRKYs and SlRIN or SlERFs found in the present study further supports their direct regulatory roles in color change during fruit ripening. As described above, SlRIN is a key regulator of fruit ripening that functions in the early stage of the process and was observed to directly interact with several genes related to color change, including *SGR1*, *PSY1* and other lycopene biosynthesis genes^22,41^. ERFs are important components for ethylene signaling and directly regulate a large set of target genes, leading to physiological changes induced by ethylene^63^. The two SlERFs found to interact with the SlER-WRKYs in the present study, SlERF7 (LeERF1)^42^ and SlERF2b (TERF2/LeERF2)^43^, reportedly function in carotenoid accumulation and ethylene biosynthesis, respectively. SlERF2b (LeERF2/SlERF.E1) was recently confirmed to be markedly up-regulated during tomato fruit ripening and to be positively regulated by SlRIN^64^.

In VIGS approach for silencing the 8 SlER-WRKY genes indicates obvious effect in the fruit ripening process, especially *SlER-WRKY 16*, *17*, *53* or 54, which present high similarities to *AtWRKY 6* and *AtWRKY53*, respectively (Figs 1 and 10b). It is of significance to find out the roles of *SlER-WRKY 16*, *17*, *53* and 54 in fruit ripening, since AtWRKY 6 and AtWRKY 53 have been found to play vital roles in leaf senescence^37,50,51^. Multiple knock-outs of genes by the recently developed CRISPR/Cas9 system will be introduced as the next step of the functional characterization of these genes^65^.

In conclusion, 12 *SlWRKY* genes were found to strongly respond to ethylene (SlER-WRKYs), indicating the involvement of WRKYs in tomato fruit ripening. Eight of the 12SlER-WRKYs were found to have the potential to directly regulate 4 genes related to color change, *SlPAO*, *SlPPH*, *SlPSY1* and *SlPDS*. These SlER-WRKYs may form complexes with each other or with other TFs and may connect to the intricate regulatory network that controls in tomato fruit ripening. Further genetic data by RNAi or CRISPR/Cas9 lines will help to confirm the function of the SlER-WRKY genes.

## Materials and Methods

### Plant material and fruit treatments

Tomato plants (*Solanum lycopersicum* var. cerasiforme) were grown in the greenhouse under long-day conditions (16-h light, 8-h dark) at a temperature of 26 °C in the light and 18 °C in the dark at the Vegetable Research Institute, Academy of Agriculture Sciences of Guangdong, Guangzhou, China. Green mature tomato fruits with uniform shape, color and size were selected and randomly divided into 3 groups of 90 fruits for each. These fruits were treated with 100 µL L^−1^ ethylene, 1 µL L^−1^ 1-MCP (1-methylcyclopropene) or air (control) for 24 h in airtight boxes at 25 °C. Afterward, the fruits of each treatment were placed evenly into 3 baskets, which served as 3 replicates, and allowed to ripen at 25 °C and 80–90% relative humidity (RH). Fruits from each replicate were randomly removed at 0, 3, 5, 7, and 9 d after treatment. Fruit respiration and ethylene production rates and firmness were measured at each time point. Fruit pericarp tissues at each time point were sampled, frozen in liquid N_2_ and stored at −80 °C to measure the contents of chlorophyll and lycopene and for gene expression analysis. The leaves and fruits at different plant developmental stages were sampled from plants in the greenhouse. Samples from 3 different plants served as 3 replicates.

### Characterization of the ripening parameters of fruits

To measure ethylene production and respiration rates, ten tomatoes randomly sampled from each treatment at each time point, were sealed in a container and held for 3 h at 25 °C. The headspace gas was collected and measured using a gas chromatograph (GC) (Model GC-17A, Shimadzu Co., Kyoto, Japan)^66^. The ethylene production and respiration rates were expressed as µL kg^−1^ h^−1^ and mg kg^−1^ h^−1^ fresh weight basis, respectively.

Fruit firmness was determined using a digital force pressure tester equipped with a 2-mm-diameter round plunger with a flat surface (Model Instron 5542, Instron Co., USA)^67^. Five fruits from each treatment at each time point were measured. Fruit firmness was expressed as mean Newtons (N).

Chlorophyll was extracted by grinding 1 g of fruit pericarp tissue in 5 mL of 80% (v/v) cold acetone and soaked for 30 min at 4 °C. For measurement of the lycopene, 2 g of fruit pericarp tissue was ground in liquid N_2_ and extracted in 5 mL of dichloroethane for 3 h at 35 °C^68^. The organic phase of both extracts was collected for detecting the absorbance at 484 and 652 nm using a spectrophotometer (UV2450, Shimadzu Co., Japan). The chlorophyll and lycopene content was expressed as μg g^−1^ fresh weight.

### RNA extraction and qRT-PCR analysis

Total RNA from the frozen pericarp tissues was extracted using a total RNA Extraction Kit (Yueyang, Beijing, China). After obtaining pure RNA, the synthesis of cDNA was carried out using a cDNA synthesis kit (PrimeScript^TM^ RT Reagent Kit) with gDNA Eraser (Perfect Real Time, Takara, Japan). The cDNA products were extracted for use in RT-qPCR.

The specific primers of the SlWRKY genes were designed based on the 3′-untranslated region by searching the SGN database (http://solgenomics.net/organism/Solanum lycopersicum/genome) or were adopted from those of the previous reports^39^. The primers of genes related to ripening and the *SlWRKY* genes used for qRT-PCR analyses are listed in Supplementary Table S3. qRT-PCR was performed using a Bio-Rad CFX96 Real-Time PCR System (Bio-Rad, USA) in a 20-μL reaction containing 10 μL of Thunderbird Mix (Toyobo, Japan), 1 μL each of the forward and reverse primers (0.25 mM), and 2 μL of diluted cDNA template. The program included an initial denaturation step at 95 °C for 3 min followed by 40 cycles of 95 °C for 15 s, 55 °C for 10 s, and 72 °C for 10 s. The Ct values for all genes were calculated using Bio-Rad CFX Manager software version 1.5. The relative expression levels of all genes were calculated and analyzed by normalizing to the Ct value of elongation factor 1 alpha (*EF-1α*)^69^ as a reference gene according the 2^−ΔΔCt^ method^70^. The values represent the means of three biological replicates.

### Subcellular localization analysis of SlWRKY proteins

The open reading frames (ORFs) of *SlWRKY16*, 17, 22, 25, 31, 33, 53 and 54 were amplified and cloned into the pCambia35tleYFP vector using a One Step Cloning Kit (Vazyme, China). The primers are listed in Supplementary Table S4. The *SlWRKYs* were expressed in frame, with *YFP* encoding the yellow fluorescent protein under the control of the cauliflower mosaic virus (CaMV) 35 S promoter. The resulting constructs were then introduced into *Agrobacterium* strain EHA105. Tobacco (*Nicotiana benthamiana*) infiltration was performed as described previously^71^. After 48 hours of infiltration, the nuclei were stained with 4,6-diamidino-2-phenylindole (DAPI), and the fluorescence signals of the DAPI-stained nuclei and the signals of the *SlWRKY-*YFP fusion proteins were imaged using an LSM710 confocal microscope (Zeiss, Germany).

### Yeast one-hybrid (Y1H) assay

Y1H assays were performed using a Matchmaker™ Gold Y1H System (Clontech, Japan). The promoters of the *SlACO1*, *SlACO3*, *SlPAO*, *SlPPH*, *SlSGR1*, *SlPSY1*, and *SlPDS* genes were cloned by an enzyme restriction method into the pAbAi vector carrying the AUR1-C gene; the primers are listed in Supplementary Table S5. The ORFs of *SlWRKY16*, 17, 22, 25, 31, 33, 53 and 54 genes were cloned into pGADT7-AD vectors; the primers are listed in Supplementary Table S6. The strains were then allowed to grow for 2–3 d at 30 °C to assess DNA–protein interactions according to previous methods^72^.

### Dual luciferase assay

Reporter constructs were generated by cloning the promoters of the *SlACO1*, *SlACO3*, *SlPAO*, *SlPPH*, *SlPSY1*, and *SlPDS* genes into *pGreenII 0800-LUC* vectors using a One Step Cloning Kit (Vazyme, China). The primers are listed in Supplementary Table S7. The ORFs of *SlWRKY16*, 17, 22, 25, 31, 33, 53 and 54 were cloned into the pCambia35tleYFP vector using the One Step Cloning Kit; the primers are listed in Supplementary Table S8. For the transient expression assay, tobacco (*Nicotiana benthamiana*) leaves were co-infiltrated with *Agrobacterium* GV3101 containing the reporter and effector vectors as described above. The ratios of enzyme activities of firefly luciferase (Luc) to renilla luciferase (Ren) were analyzed using a dual luciferase reporter assay system (Promega, USA) and a Luminoskan Ascent Microplate Luminometer (Thermo, USA). At least six transient transformations were carried out for each assay, and the assays were repeated twice.

### Yeast two-hybrid (Y2H) and transcriptional activity assays

Y2H assays were performed using the Matchmaker™ Gold Y2H System (Clontech, Japan). The full-length coding regions of *SlWRKY16*, *SlWRKY*17, *SlMADS-RIN*, *SlERF2b*, and *SlERF7* were cloned into the pGBKT7 vector containing the GAL4 DNA-binding domain (DBD) to create different baits using a One Step Cloning Kit (Vazyme, China). The primers are listed in Supplementary Table S9. At the same time, the ORFs of *SlWRKY16*, *17*, 22, 25, 31, 33, 53, and 54 were cloned into the pGADT7 vector in-frame with the GAL4 activation domain (AD) to create relevant prey; the primers are listed in Supplementary Table S8. Different pairs of bait and prey constructs were co-transformed into the Y2HGold yeast strain.

The transcriptional activities of the selected 8 SlWRKY TFs were also detected in the Y2HGold yeast. The ORFs of the SlWRKY16, 17, 22, 25, 31, 33, 53 and 54 genes were cloned into the pGBKT7 vector. The primers for the cloning are listed in Supplementary Table S8. Transformed yeast cells were then grown on SD/-Trp or SD/-Trp-Ade-His medium at 30 °C for 3–4 d. An assay of α-galactosidase activity was performed using X-α-gal^73^.

### Bimolecular fluorescence complementation (BiFC) assay

For the BiFC assay, the ORFs of *SlWRKY16*, *SlWRKY17*, *SlRIN*, *SlERF2b*, and *SlERF7* were cloned into the pSAT6-cEYFP-C1-B vector for in-frame expression of these genes with the C-terminus of *YFP*, and the ORFs of *SlWRKY17*, 33, 53 and 54 were cloned into the pSAT6-n (1–174) EYFP-C1 vector for in-frame expression with the N-terminus of *YFP*. The primers are listed in Supplementary Table S10. Pairs of the above pSAT6-cEYFP and pSAT6-nEYFP constructs were co-transformed into Arabidopsis protoplasts using the PEG transfection method. Protoplast isolation was performed as described previously^74^. After obtaining the transfected cells, the results were imaged using a confocal microscope as described above.

### Virus-induced *SlWRKY* gene silencing in tomato

For the VIGS experiment, pTRV1 and pTRV2-LIC (used for free cloning) vectors were employed. The plasmid construction was performed as described in Dong *et al*.^75^, and the primers of *SlWRKY* genes were listed in Supplementary Table S11. The Agrobacterium tumefaciens strain GV3101harboring pTRV1, pTRV2-LIC, or pTRV2-LIC-SlWRKYs (target genes) were incubated. Tomato fruit infiltration was performed as described by Orzaez *et al*.^76^, and Li *et al*.^42^, with little modifications. The *Agrobacterium* strain GV3101 containing pTRV1 and pTRV2-LIC-SlWRKYswere mixed in a 1:1 ratio and infiltrated into the mature green fruit tissue through the stylar apex with a 1-mL needle-less syringe. The fruit that infiltrated with pTRV1 and pTRV2-LIC without the gene fragments was used as control. Then tomato fruit were allowed to ripen at 25 °C and 80–90% RH for 14 days after infiltration. Infiltration for invidious gene was performed 3 times with 10 fruits for each repeat. The red color a* values of each fruit were measured by 14 days after infiltration. RNA was isolated from the pericarp tissue of each fruit and the transcription levels of the *SlWRKY* genes were analyzed by real-time PCR as described above. The transcription levels and a* values of 6 representative fruit with efficient silencing were subjected for statistics analysis.

## Electronic supplementary material

Supporting Information
