# Supplementary material for: Regulation of ethylene-responsive SlWRKYs involved in color change during tomato fruit ripening
Source: Sci Rep. 2017 Nov 30;7:16674. doi: 10.1038/s41598-017-16851-y (PMC5709409; doi:10.1038/s41598-017-16851-y)
Supplement: Supplementary file 1 — Supporting Information [file 41598_2017_16851_MOESM1_ESM.pdf]

Supplementary Information

Regulation of ethylene-responsive *SlWRKY*s involved in color change during tomato fruit ripening

Ling Wang[1, 2, 4 †], Xue-lian Zhang[1, 3, †], Lu Wang[1, 2, 4], Yanan Tian[1, 3], Ning Jia[1, 2, 4], Shuzhen Chen[1, 3], Ning-bo Shi[1, 2, 4], Xuemei Huang[1, 2, 4], Chu Zhou[1, 3], Yaowen Yu[1, 2, 4], Zhao-qi Zhang[1, 2, 4*], Xue-qun Pang[1, 3, 4*]

[1] State Key Laboratory for Conservation and Utilization of Subtropical Agro-bioresources, South China Agricultural University, Guangzhou, 510642, China; [2] College of Horticulture, South China Agricultural University, Guangzhou, 510642, China; [3] College of Life Sciences, South China Agricultural University, Guangzhou, 510642, China; [4] Guangdong Provincial Key Laboratory of Postharvest Science of Fruits and Vegetables, Guangzhou 510642, China

*Authors for Correspondence:

Xue-qun Pang, e-mail: xqpang@scau.edu.cn

Zhao-qi Zhang, e-mail: zqzhang@scau.edu.cn

[†] These authors contributed equally to the paper.

Running Title: Regulation of SlER-WRKYs during tomato color chang

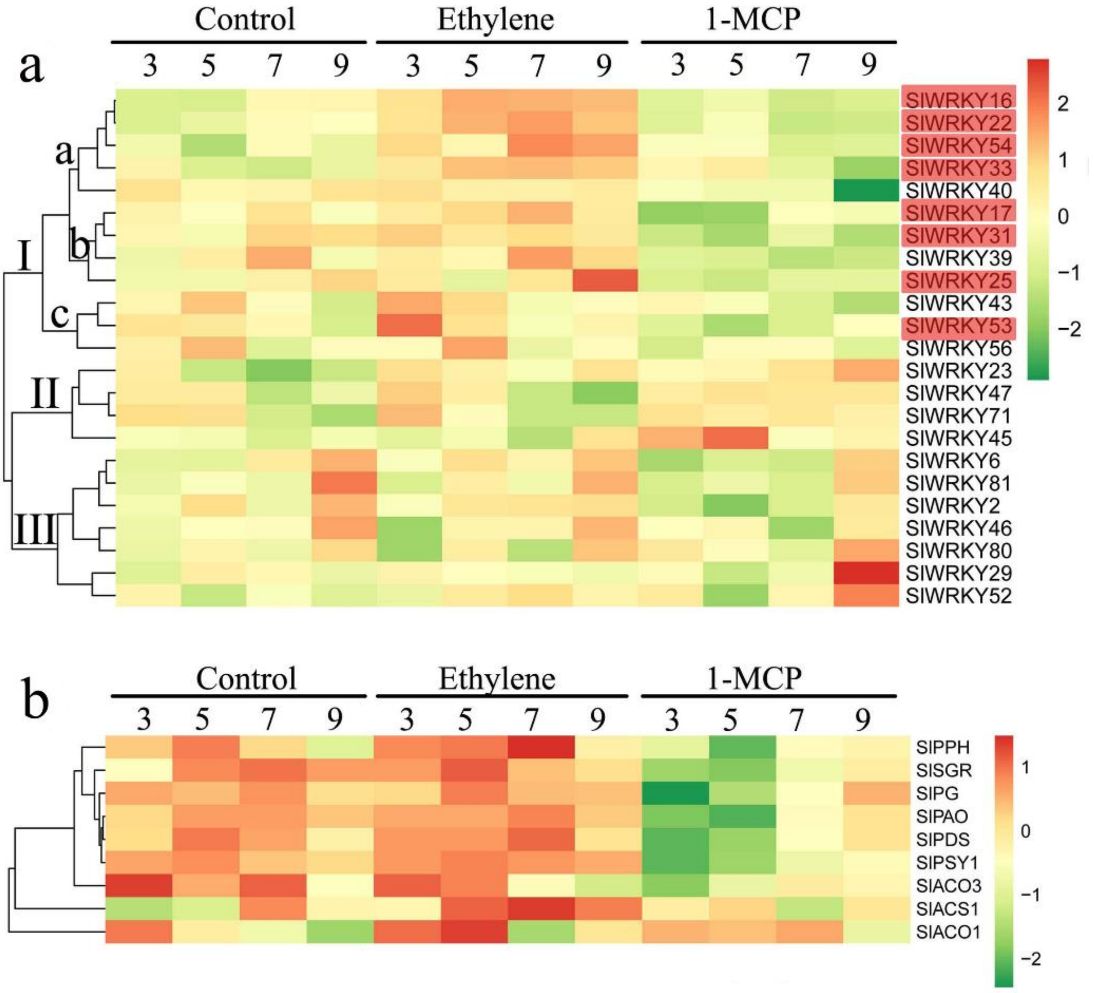

Supplementary Figure 1. Hierarchical cluster of transcript levels for color change related genes and the selected *SlWRKYs* during tomato fruit ripening in response to ethylene and 1-MCP. The complete linkage hierarchical clustering of these genes were performed by heatmap according to the ClustVis program available at http://biit.cs.ut.ee/clustvis/. The cluster was generated using the distance of the coefficient correlation of gene expression values by Pearson clustering algorithm method. (a) Hierarchical cluster of transcript levels for the 23 selected *SlWRKY* genes. (b) Hierarchical cluster of transcript levels for 5 color changerelated genes with the reference of 3 ethylene biosynthesis genes (*ACS1*, *ACO1* and *ACO3*) and a cell wall metabolism gene (*PG*). Red and green boxes respectively indicate higher and lower levels of expression compared to the levels before the treatments at day 0. The color density is directly proportional to the expression levels. The transcript levels were detected by Real-time RT-PCR in the samples collected from the control, ethylene or 1-MCP treated fruits at the indicated time points as described in Fig. 2. The labels for 8 ethylene responsive SlWRKYs in cluster I and selected for further analysis were highlighted in red.

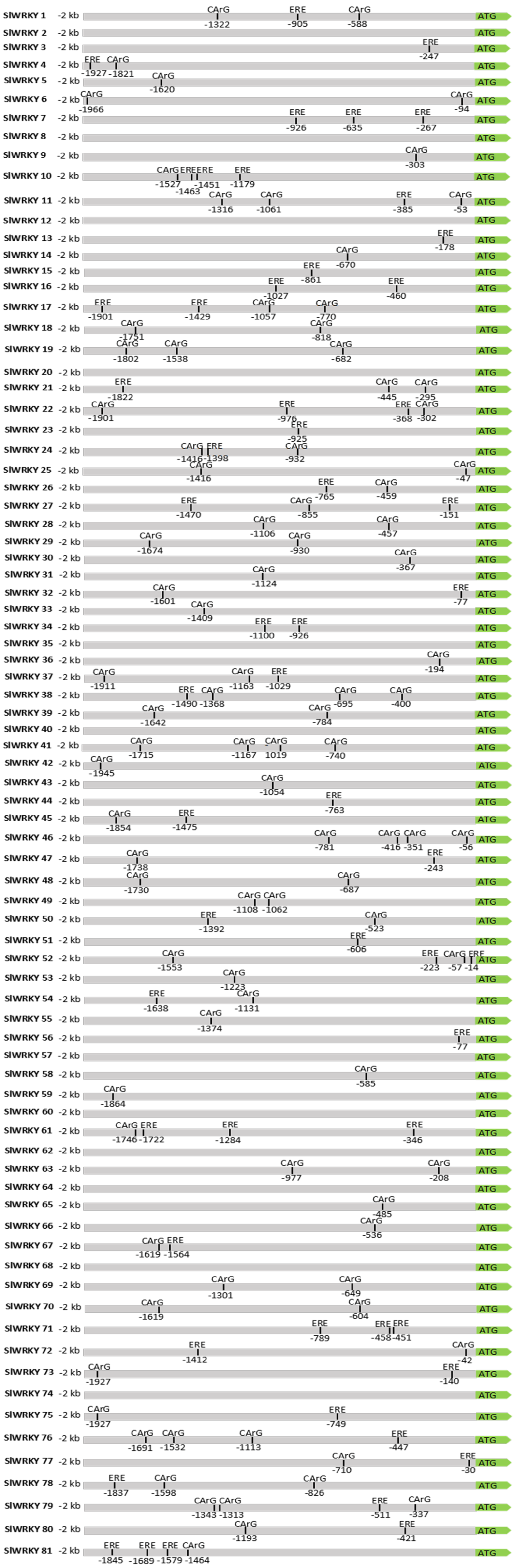

Supplementary Figure 2. The ethylene-response elements (ERE) and RIN-binding sites (known as CArG box) promoter regions of 81 SlWRKY genes. The conserved cis-regulatory elements of ERE and CArG were predicted at http://bioinformatics.psb.ugent.be/webtools/plantcare/html/). Accordingly, among selected 23 SlWRKY genes, *SlWRKY16, 17, 22, 23, 45, 47, 52, 54, 71, 80,* and *81* existed ethylene-response elements, *SlWRKY6, 17, 22, 25, 29, 31, 33, 39, 45, 46, 47, 52, 53, 54, 80,* and *81* existed CArG box.

a

b

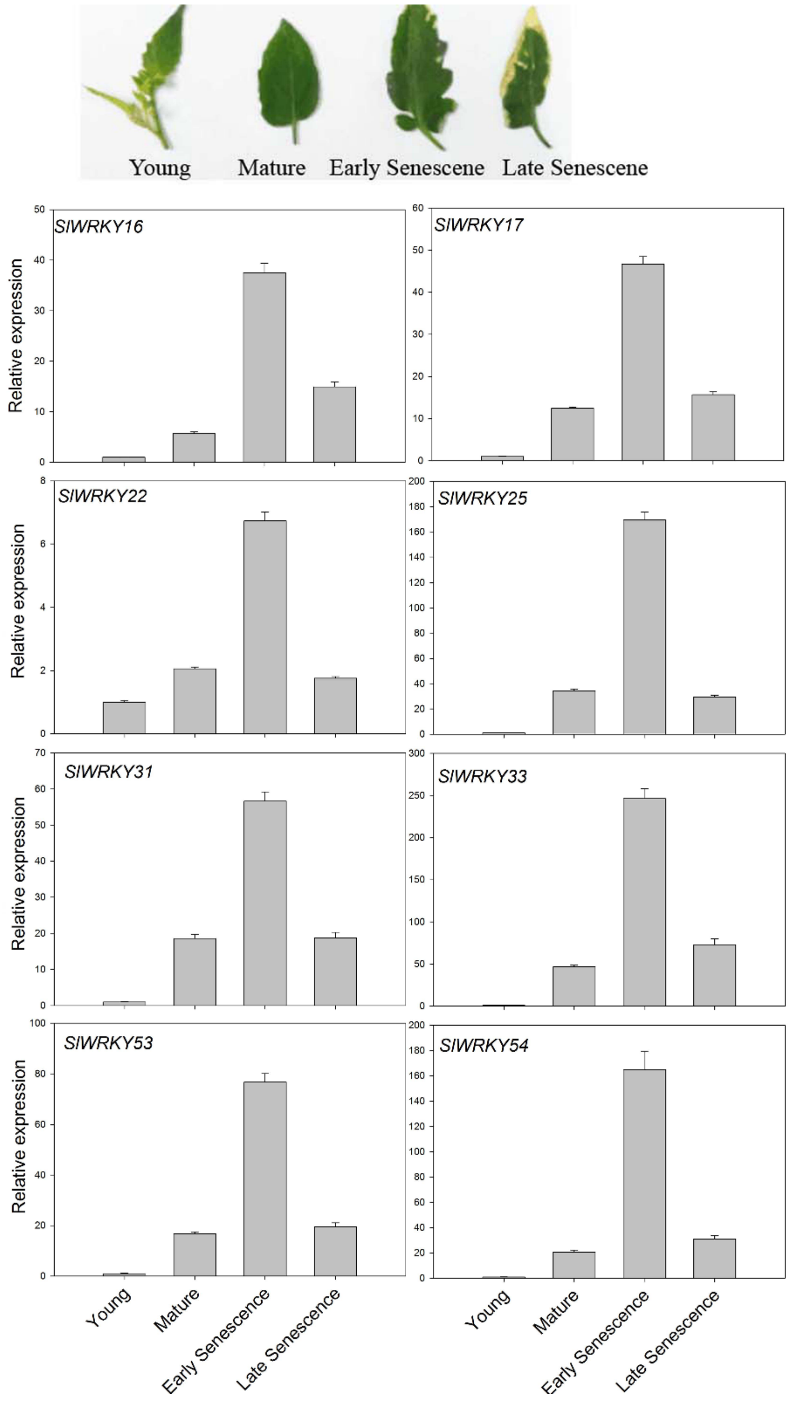

Supplementary Figure 3. Transcription profiling of the 8 *SlER-WRKY* genes during tomato leaf development. (a) Leaf samples were collected from young, mature, early senescent and late senescent leaves. (b) The transcription profiling of the 8 *SlER-WRKY* genes at these leaf developmental stages were as described in Supplementary Fig. S1a.

a

b

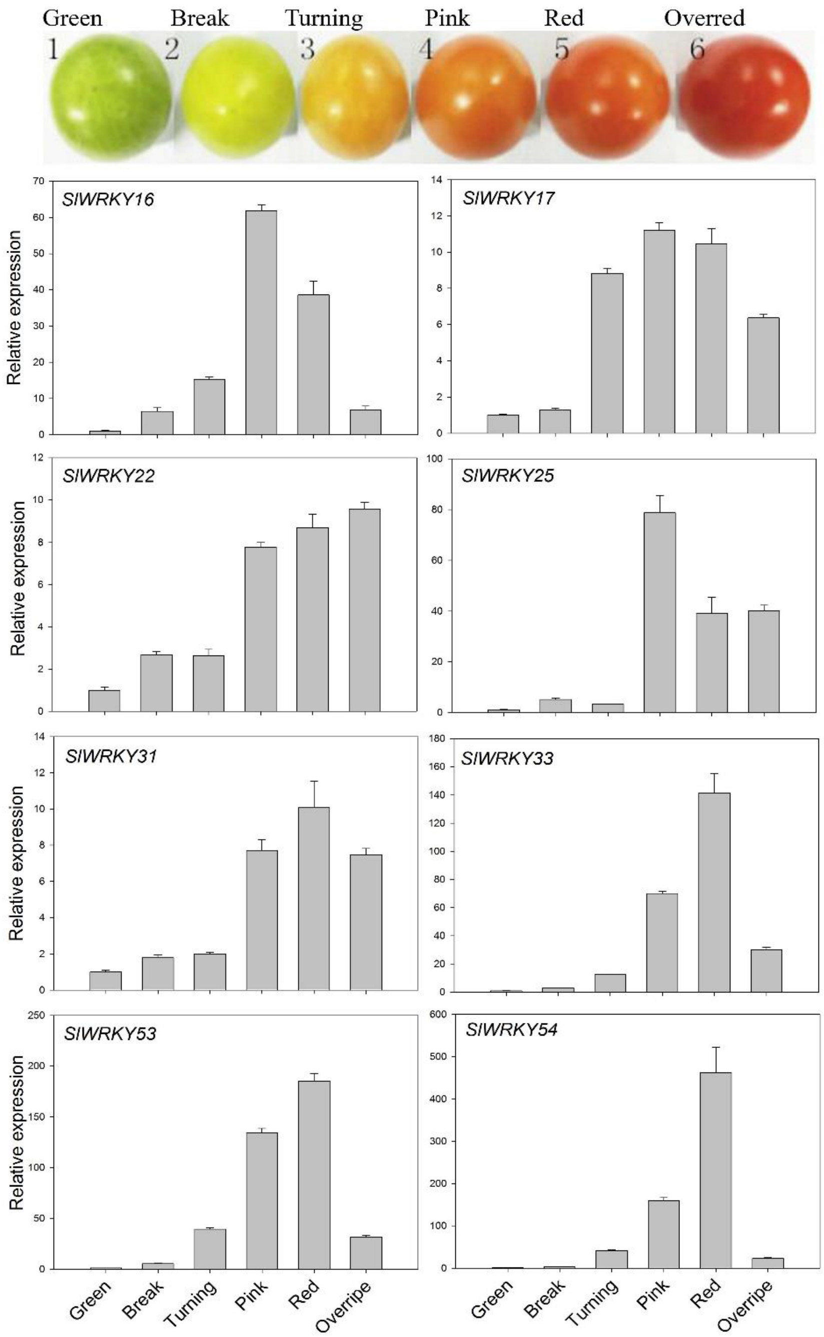

Supplementary Figure 4. Transcription profiling of the 8*SlER-WRKY* genes in the tomato fruit ripening in plants. (a) Fruit pericarp samples were collected at green mature, breaking, turning, pink red and overripe stages during the fruit ripening in plants. (b) The transcription profiling of the 8 *SlER-WRKY* genes at these stages were as described in Supplementary Fig. S1a.

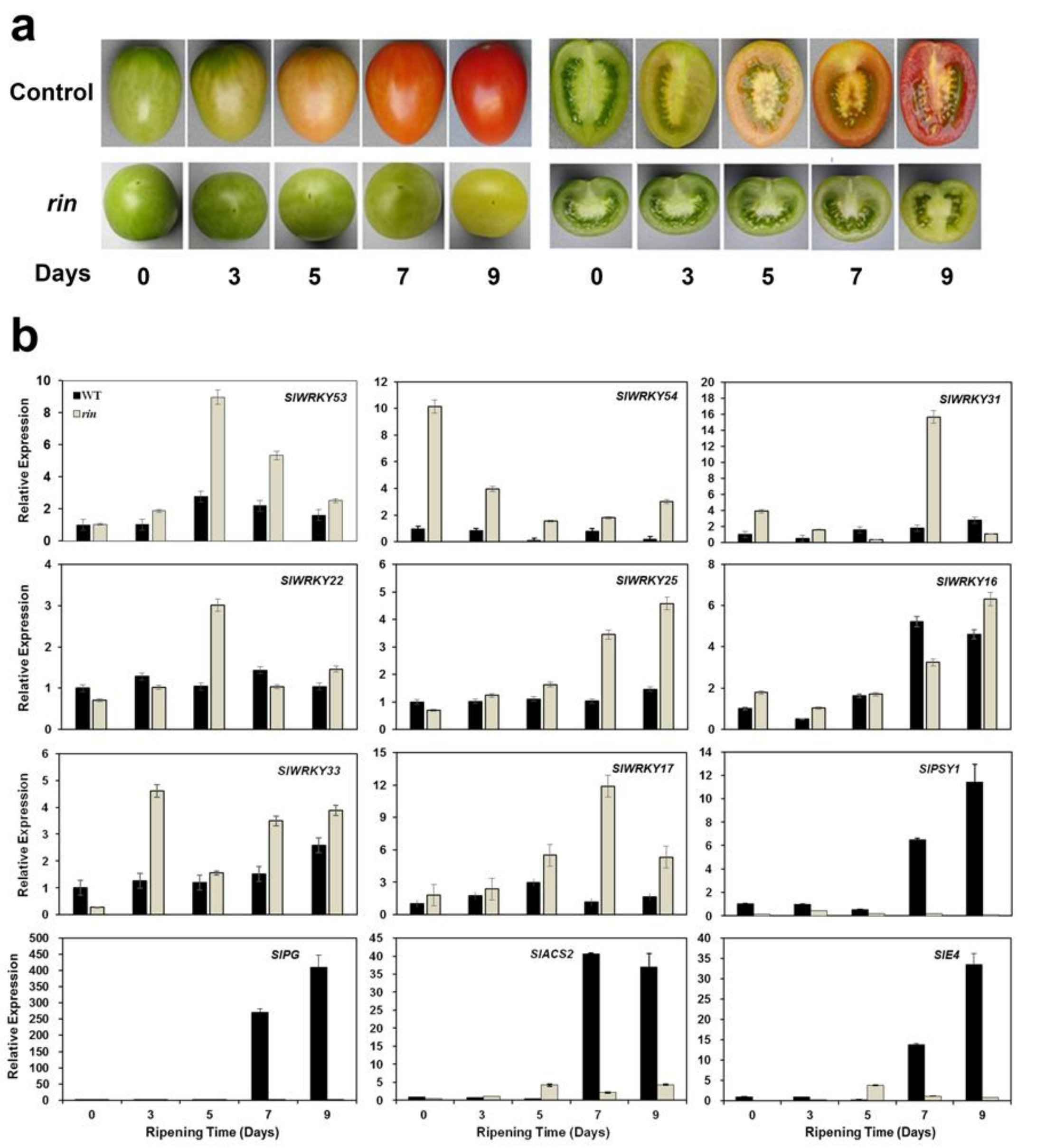

Supplementary Figure 5. Transcription profiling of the 8 *SlER-WRKY* genes and 4 fruit ripening related genes in wild type (WT) and *rin* mutant tomato fruit. The ripening inhibitor (*rin*) mutant (NIL LA3754) was a gift from Tomato Genetics Resource Center (USA). The *rin* mutant is a single gene mutation, fruit attain full size and produce viable seed; however, they are arrested in the fruit ripening [1]. The 8 ethylene-responsive *SlWRKY* (*SlER-WRKY*) genes were identified based on the gene transcription profiles in Fig. 4 and Supplementary Fig. S1a; the 4 typical fruit ripening related genes were selected according to Martel et al. (2011) [2]. (a) Fruit pericarp samples were collected at green mature, breaking, turning, pink red and overripe stages during the fruit ripening in plants. (b) The transcription profiling of the 8 *SlER-WRKY* genes and 4 fruit ripening related genes at these stages.

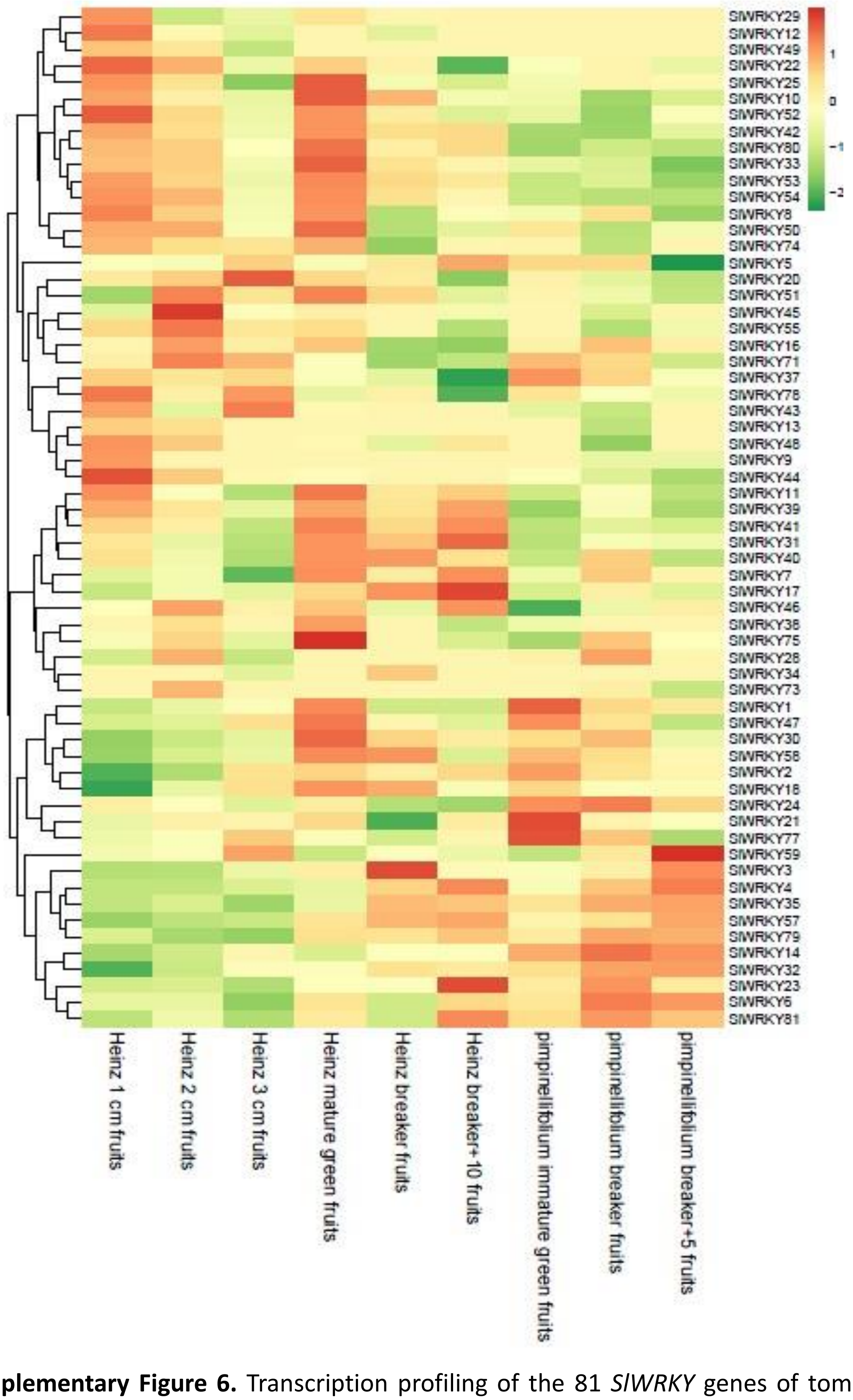

Supplementary Figure 6. Transcription profiling of the 81 *SlWRKY* genes of tomato fruit. The gene expression of fruit in different developmental process were obtained from the RNA-seq data at http://ted.bti.cornell.edu/cgi-bin/TFGD/digital/home.cgi.

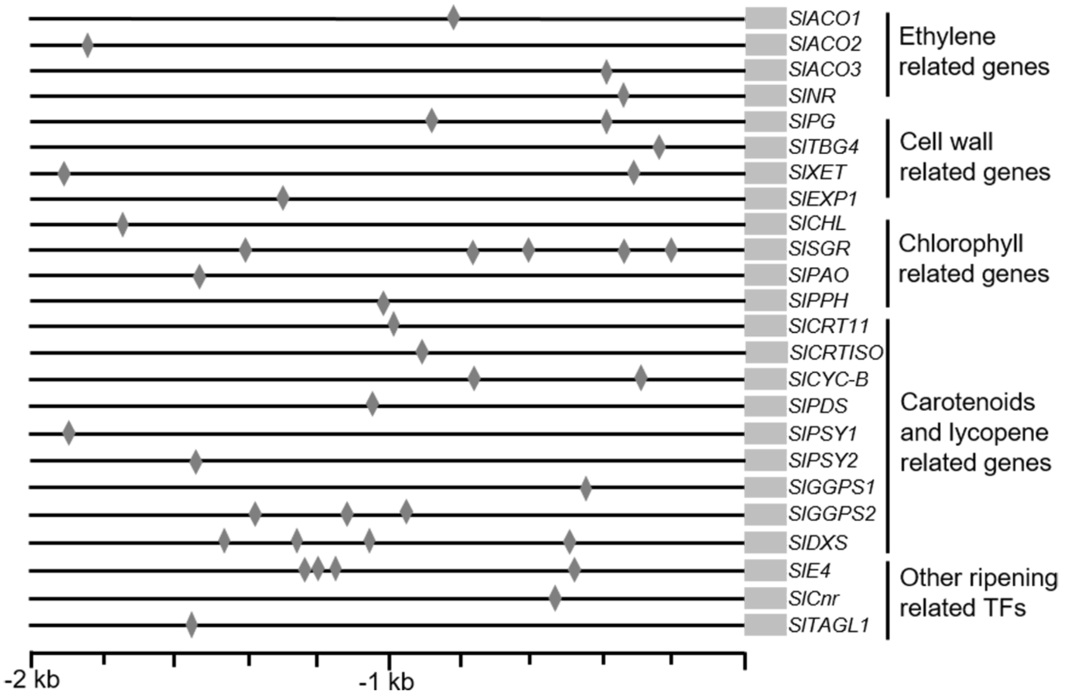

Supplementary Figure 7. The WRKY-binding sites (known as W-box) promoter regions of tomato fruit ripening process related genes and TFs. In addition to the selected 9 ripening related genes, W-box binding sites in promoter region of other genes that involved in ethylene, cell wall, chlorophyll, carotenoids and lycopene metabolisms, as well as several ripening regulatory TFs were predicted. It suggests that SlWRKYs may be involved in regulation of fruit ripening. The conserved cis-regulatory elements were predicted at http://bioinformatics.psb.ugent.be/webtools/plantcare/html/.

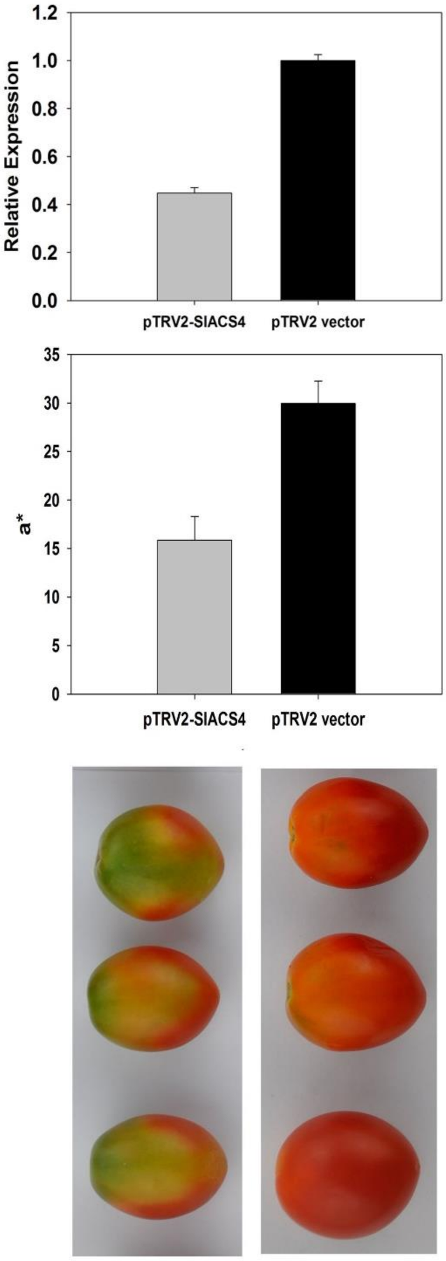

Supplementary Figure 8. VIGS based transient gene silencing of an ethylene bio-synthesis gene, *SlACS4* as a marker. Transcription profiling of the 8 *SlER-WRKY* genes in the tomato fruit ripening in plants. The efficiency of the silencing was first confirmed by evaluation of silencing, using the same approach. The relative expression of the *SlACS4* gene, the red color a* values and the appearance of the tomato fruits at 14 days after infiltration were shown in a same column.

Table S1. Correspondence between the unified nomenclature of the SlWRKY gene family and their Solyc identifiers. The relevance of other names as well as the function proposed in the literature are also listed.

New Names[a]	Solyc Identifier	Other Names	Reported function	References
SlWRKY4	Solyc05g012770.2.1	SpWRKY2	Pathogen resistance	[3]
SlWRKY31	Solyc06g066370	SlDW1	Disease resistance	[4]
SlWRKY40	Solyc06g068460.2	SlWRKY1	Disease resistance	[5]
SlWRKY73	Solyc03g113120.2	SlWRKY72a	Immune responses	[6]
SlWRKY74	Solyc06g070990.2	SlWRKY72b	Immune responses	[6]
SlWRKY80	Solyc03g095770.2	SlWRKY70	Insect infestation	[7]

[a] The new names are issued according to Huang et al.[8], and the Solyc chromosome identifier issued by ITAG 2.40 reference annotation [9].

Table S2. The sequences and main functions of WRKY-TFs that have been reported

Gene Name	Species	References	Gene Bank	Function
AtWRKY6	Arabidopsis	[10,11]	NP_564792.1	Senescence
AtWRKY8	Arabidopsis	[12]	NP_199447.1	Induced by ACC
AtWRKY18	Arabidopsis	[13]	NP_567882.1	Senescence
AtWRKY22	Arabidopsis	[14]	NP_192034.1	Senescence
AtWRKY30	Arabidopsis	[15]	NP_568439.1	Senescence
AtWRKY33	Arabidopsis	[16]	NP_181381.2	Combine with promoter of *ACS*
AtWRKY53	Arabidopsis	[17-19]	NP_194112.1	Senescence
AtWRKY54	Arabidopsis	[15]	NP_181607.1	Senescence
AtWRKY57	Arabidopsis	[20]	NP_177090.1	Senescence
AtWRKY70	Arabidopsis	[15,21]	NP_191199.1	Senescence
AtWRKY75	Arabidopsis	[22]	NP_196812.1	Senescence
OsWRKY23	*Oryza sativa*	[23]	NP_001044163.1	Senescence
OsWRKY71	*Oryza sativa*	[24]	NP_001046094.1	Induced by ACC
OsWRKY80	*Oryza sativa*	[25]	NP_001063497.1	Senescence
GbWRKY1	*Gossypium barbadens*	[26]	AFH35047.1	Induced by ACC
GhWRKY40	*Gossypium hirsutum*	[27]	AGX24945.1	Response of

				ethylene signal
BnWRKY1	*Brassica napus* L	[28]	ACI14383.1	Response of ethylene signal
BnWRKY28	*Brassica napus* L	[28]	ACI14393.1	Response of ethylene signal
BnWRKY32	*Brassica napus* L	[28]	ACI14396.1	Response of ethylene signal
BnWRKY33	*Brassica napus* L	[28]	ACI14397.1	Response of ethylene signal
BnWRKY40	*Brassica napus* L	[28]	ACI14400.1	Response of ethylene signal
BnWRKY45	*Brassica napus* L	[28]	ACH99806.1	Response of ethylene signal
BnWRKY69	*Brassica napus* L	[28]	ACI14405.1	Response of ethylene signal
BnWRKY70	*Brassica napus* L	[28]	ACI14407.1	Response of ethylene signal
BnWRKY75	*Brassica napus* L	[28]	ACI14409.1	Response of ethylene signal

Table S3. The primers of *SlWRKYs* and fruit ripening related genes for Real-time PCR

Gene Name	Gene ID	Primer (5' -3')	Length	Note
SlWRKY2	Solyc07g066220.2.1	ACCTACGAAGGGAAGCACA<br>CGATTTGACAGGCAACCTTC	176bp	1
SlWRKY6	Solyc02g080890.2.1	TCAACCAAGCAAATCCTC<br>TGATGGGTGGTGATGATG	179bp	
SlWRKY15	Solyc10g005680.1.1	GAAGAACAGCAGGCGTTAC<br>AGTTGAATCCGGTCATAGA	117bp	
SlWRKY16	Solyc02g032950.2.1	TGG CAT CGA CTA CAT CAG<br>TTA CAG TGG GAA ACG GAG	159bp	
SlWRKY17	Solyc07g051840.2.1	ACAACATCCTAGCAAATC C<br>TTCACCGTGTCAGCTACA	178bp	
SlWRKY22	Solyc01g095100.2.1	GAG AAG CAA GAA AGC AGC A<br>TAT CGG AAA AGC AAT CTC C	169bp	
SlWRKY23	Solyc01g079260.2.1	TGGAGATGCTGATGGGGAAG<br>AAACGCAAATCTCGGCTCTT	118bp	1
SlWRKY25	Solyc10g011910.2.1	TAAAGGTTCCCCATACCCAAG<br>GTTTTTTGGCGGCTGATTC	183bp	1
SlWRKY29	Solyc08g081610.2.1	TTCTCCAGAAACACTTATGATCG<br>AGACCAGCAAAGAAATCTCCA	144bp	
SlWRKY31	Solyc06g066370.2.1	ACAACCTATGAAGGGAAGCACA<br>AGGGTGCTCCCATTTCAGAC	175bp	1

SlWRKY33	Solyc09g014990.2.1	CGGTTAGGAAGCATGTGGAA<br>ATCTGGAGGGTGATTGGTGG	265bp	
SlWRKY39	Solyc03g116890.2.1	GCGGTAATGCCAAGACAAAC<br>TCAGTTCCTGGTGATTTACGC	134bp	
SlWRKY40	Solyc06g068460.2.1	AGGACCAACCATTACTCTCG<br>CTGCTGGACTTTTGTTTACC	298bp	
SlWRKY45	Solyc08g067360.2.1	GCCCAGTCAAGAAGAAGGTA<br>GAAAGTAGGGCTGCTGGTTA	233bp	
SlWRKY46	Solyc08g067340.2.1	CCAACCACCTCAAGCTGAA<br>AAGACGCCATTTGTTCGACT	131bp	
SlWRKY52	Solyc03g007380.1.1	GGTGCTAAACATCCGAGAG<br>CTGACACTAGCACCACCAC	158bp	
SlWRKY53	Solyc08g008280.2.1	CACATACCGAGGCTCCCATAA<br>CCTGTTGGATAAACGGCTTGG	105bp	
SlWRKY54	Solyc08g082110.2.1	CAACAAAGCAAGTGCAGAGG<br>TGGTTGTGTAGCAGCAGCA	110bp	
SlWRKY56	Solyc08g081630.1.1	ATTTCATACAAGGAGCACAG<br>CATTAGCTTCTCACAAGGGT	220bp	
SlWRKY71	Solyc02g071130.2.1	GTAGCGTCGCAAGGATGTTG<br>TGCTCAAAGCCTCATGGTTCT	271bp	
SlWRKY80	Solyc03g095770.2.1	AAGAAGGAGAAGCAAGACCGTA<br>TCTTGATCGTACTTGTGGGTGC	179bp	1

SlWRKY81	Solyc09g015770.2.1	CTCAGATTTGGTGGATGATGGT<br>AAATCTTTGTGGGTTGTCTTGG	163bp	1
SlSGR	Solyc08g080090.2.1	AAAATGGGACCATCCAACAA<br>GCTGCTTCCACAAACCCTAT	137bp	
SlPPH	Solyc01g088090.2.1	CCCATGATGAAGTCCCAGAG<br>GGGAGAGGCTTTCCATGTTT	221bp	
SlPAO	Solyc11g066440.1.1	TGGATTAGCATACATTCTACACGAA<br>TTGTGTTTTGTGCTGTTTCTGA	158bp	
SlPSY1	Solyc03g031860.2.1	CAAATGGGACAAGTTTCATGGA<br>TTCCTATGCCTCGATGAATCAA	70bp	3
SlPDS	Solyc03g123760.2.1	AAGGCGCTGTCTTATCAGGAAA<br>TAAACTACGCTTGCTTCCGACA	106bp	3
SlPG	Solyc10g080210.1.1	TATTACTTGTGGTCCAGG<br>ATCCTAACTCCATTTTCG	126bp	
SlACO1	Solyc07g049530.2.1	CACTAACGGGAAGTACAAGA<br>ACCATACATAAGAAGAGCAA	269bp	2
SlACO2	Solyc12g005940.1.1	GCATCCTTCTACAATCCAGGA<br>CATGTAGTAGGGACGCACA	332bp	2
SlACO3	Solyc07g049550.2.1	GAGCGTGATGCACAGAGTGA<br>CAATCACACACACATACACCA	349bp	2
SlACS1	Solyc08g081540.2 1	CGGGCTAGTTTCAACTCAGA<br>CAACAACAACAAATCTAAGCCA	566bp	2

SlACS2	Solyc01g095080.2.1	AAGCGCGATGAGGTTAGGTA<br>AAAGTGGACGCAAATCCATC	119bp	4
SlE4	Solyc03g111720.2.1	GACCACTCTAAATCGCCAGG<br>TTCCTGAGCGGTATTGCTTT	53bp	4
EF-1α	Solyc06g005060.2.1	AGATGGTCAGACCCGTGAAC<br>TGGAGTACTTGGGGGTGGTA	104bp	3

Note：1 The primer quoted from the [8]

2 The primer quoted from the [29]

3 The primer quoted from the [30]

4 The primer quoted from the [2]

Table S4. The primers used for construction pH7WGY2-SlWRKYx vector

Name of Genes	Primers（5′-3′）
SlWRKY16-pH7WGY2-For	TTACGAATTTCGACCTGCAGatggataaaggatggggacttac
SlWRKY16-pH7WGY2-Rev	CAGTTGGAATTCTAGActactgttcttttccaggacc
SlWRKY17-pH7WGY2-For	TTACGAATTTCGACCTGCAGatggccaaaggaagtggac
SlWRKY17-pH7WGY2-Rev	CAGTTGGAATTCTAGAgtttgctggaaaactcga
SlWRKY22-pH7WGY2-For	TTACGAATTTCGACCTGCAGatggaagaggattgggatct
SlWRKY22-pH7WGY2-Rev	CAGTTGGAATTCTAGAaacgccgcctgcgg
SlWRKY25-pH7WGY2-For	TTACGAATTTCGACCTGCAGatggaaagctacaaagacatta
SlWRKY25-pH7WGY2-Rev	CAGTTGGAATTCTAGAacgaccgcctgccgc
SlWRKY31-pH7WGY2-For	TTACGAATTTCGACCTGCAGatggctgcttcaagtttctc
SlWRKY31-pH7WGY2-Rev	CAGTTGGAATTCTAGAgcaaagcaatgactcc
SlWRKY33-pH7WGY2-For	TTACGAATTTCGACCTGCAGatggcttcttcaggtggaaat
SlWRKY33-pH7WGY2-Rev	CAGTTGGAATTCTAGAgttaaggaaagagctgaag
SlWRKY53-pH7WGY2-For	TTACGAATTTCGACCTGCAGatggattgtgcatcaaactg
SlWRKY53-pH7WGY2-Rev	CAGTTGGAATTCTAGAtgagaaaaatttggggtt
SlWRKY54-pH7WGY2-For	TTACGAATTTCGACCTGCAGatggattgtggattcaattatga
SlWRKY54-pH7WGY2-Rev	CAGTTGGAATTCTAGAtctgaaaaaatcagagaaatt

Note： The letters of lowercases are linearized homologous sequences ends of pH7WGY2 vectors.

Table S5. The primers used for construction pAbAi-SlWRKYx vector for Y1H assay

Name of genes	Primers(5′ -3′)	Enzyme sites
SlSGR-p-pAbAi-For	CGGggtaccGTTTTCAACCTCTTAATTTTGGCAG	*Kpn* Ⅰ
SlSGR-p-pAbAi-Rev	ACGCgtcgacAAAACTCCTTGAATCTTGGAGTTCC	*Sal* Ⅰ
SlPPH-p-pAbAi-For	CGGggtaccATCATCCCAAGAAGCCCCTTTTGAT	*Kpn* Ⅰ
SlPPH-p-pAbAi-Rev	ACGCgtcgacCATCCCAGAGTCAGCTTTCTAAAATCT	*Sal* Ⅰ
SlPAO-p-pAbAi-For	CCCaagcttTGTGACCTTTGAACTGTTATGTAG	*Hind* Ⅲ
SlPAO-p-pAbAi-Rev	CCctcgagTATTGAGTTAATCAATGTTTTGTGT	*Xho* Ⅰ
SlPSY1-p-pAbAi-For	CGGggtaccGGCTAAATCGAAAATTGAATCGTT	*Kpn* Ⅰ
SlPSY1-p-pAbAi-Rev	ACGCgtcgacTCTGAGCAAGAAAACCTTGGTTGT	*Sal* Ⅰ
SlPDS-p-pAbAi-For	CGGggtaccCGTCTAATATCTGGTGTTCTTCTC	*Kpn* Ⅰ
SlPDS-p-pAbAi-Rev	ACGCgtcgacCAGTAAAACCTGCCATAAGGATAT	*Sal* Ⅰ
SlACO1-p-pAbAi-For	CCCaagcttTTTGGTTAGAGGGAGAATTTGTG	*Hind* Ⅲ
SlACO1-p-pAbAi-Rev	CCctcgagTGATGTACAAATAATGAGGCTTTGA	*Xho* Ⅰ
SlACO3-p-pAbAi-For	CCCaagcttTCAAGTCAACGGATAGAAATTTCA	*Hind* Ⅲ
SlACO1-p-pAbAi-Rev	CCGctcgagGAAAAGAGTGATTCTTTGAAAGG	*Xho* Ⅰ

Table S6. The primers used for construction pGADT7-AD-WRKYx vector

Name of Genes	Primers（5′ -3′ ）	Enzyme sites
SlWRKY16-AD-For	CGCggatccATATGGATAAAGGATGGGGACTT	*BamH* I
SlWRKY16-AD-Rev	CgagctcGCTACTGTTCTTTTCCAGGACCA	*Sac* I
SlWRKY17-AD-For	CGCggatccATGGCCAAAGGAAGTGGACTCT	*BamH* I
SlWRKY17-AD-Rev	CgagctcTCAGTTTGCTGGAAAACTCGAAT	*Sac* I
SlWRKY22-AD- For	CGCggatccATATGGAAGAGGATTGGGATCTAC	*BamH* I
SlWRKY22-AD-Rev	CCGCCGctcgagCTCAAACGCCGCCTGCGG	*Xho* I
SlWRKY25-AD-For	GGGAATTCcatatgATGGAAGAAGATTGGGATCT	*Nde* I
SlWRKY25-AD-Rev	CCGCCGctcgagCACTACGGCGGCAGGCGGTCGTTGA	*Xho* I
SlWRKY31-AD-For	CGCggatccATGGCTGCTTCAAGTTTCTCT	*BamH* I
SlWRKY31-AD-Rev	CgagctcTCAGCAAAGCAATGACTCCATA	*Sac* I
SlWRKY33-AD-For	GGAATTCcatatgATGGCTTCTTCAGGTGGAAAT	*Nde* I
SlWRKY33-AD-Rev	CCGctcgagCTCAGTTAAGGAAAGAGCTGAAG	*Xho* I
SlWRKY53-AD-For	GGGAATTCcatatgATGGATTGTGCATCAAACTGG	*Nde* I
SlWRKY53-AD-Rev	CCGctcgagGTCATGAGAAAAATTTGGGGTT	*Xho* I
SlWRKY54-AD-For	GGAATTCcatatgATGGATTGTGGATTCAATTATG	*Nde* I
SlWRKY54-AD-Rev	CCGctcgagCTTATCTGAAAAAATCAGAGAAAT	*Xho* I

Table S7. The primers used for construction *pGreenII 0800-LUC* vector

Name of Genes	Primers（5′-3′）
SlACO1-LUC-For	gtcgacggtatcgataagcttGTAGACTAATGTCTGACGTTTAAC
SlACO1-LUC-Rev	cgctctagaactagtggatccTGATGTACAAATAATGAGGCTTTGA
SlACO2-LUC-For	gtcgacggtatcgataagcttCTTTTCACACTTGTTTGAAGCCAT
SlACO2-LUC-Rev	cgctctagaactagtggatccAAGATTGAGGCTTTGTTTGGGTATT
SlACO3-LUC-For	gtcgacggtatcgataagcttATGATACGTCGAAAATTCAAACG
SlACO3-LUC-Rev	cgctctagaactagtggatccGAAAAGAGTGATTCTTTGAAAGG
SlPAO-LUC-For	gtcgacggtatcgataagcttTGTGACCTTTGAACTGTTATGTAG
SlPAO-LUC-Rev	cgctctagaactagtggatccTATTGAGTTAATCAATGTTTTGTGT
SlPDS-LUC-For	gtcgacggtatcgataagcttCGTCTAATATCTGGTGTTCTTCTC
SlPDS-LUC-Rev	cgctctagaactagtggatccGGTTCTGCACAATTAAGCAAATA
SlPG-LUC-For	gtcgacggtatcgataagcttTAAGACAAAGGCCTTAAAGGACA
SlPG-LUC-Rev	cgctctagaactagtggatccCATGAGCAAATTCTTGTTCAAGAA
SlPPH-LUC-For	gtcgacggtatcgataagcttTCATCCCAAGAAGCCCCTTTTG
SlPPH-LUC-Rev	cgctctagaactagtggatccATCCCAGAGTCAGCTTTCTAAAATCT
SlPSY1-LUC-For	gtcgacggtatcgataagcttGGCTAAATCGAAAATTGAATCGTT
SlPSY1-LUC-Rev	cgctctagaactagtggatccTCTGAGCAAGAAAACCTTGGTT
SlSGR-LUC-For	gtcgacggtatcgataagcttGTTTTCAACCTCTTAATTTTGGCAG
SlSGR-LUC-Rev	cgctctagaactagtggatccACTCCTTGAATCTTGGAGTTCC

Note: The letters of lowercases are linearized homologous sequences ends of *pGreenII 0800-LUC* vectors.

Table S8. The primer used for construction *pCambia35tlegyfps* vector

Name of Genes	Primers（5' -3' ）
SlWRKY16-pCambia-For	ttacgaatttcgacctgcagATGGATAAAGGATGGGGACTTA
SlWRKY16- pCambia-Rev	cagttggaattctagaCTACTGTTCTTTTCCAGGACC
SlWRKY17- pCambia-For	ttacgaatttcgacctgcagATGGCCAAAGGAAGTGGAC
SlWRKY17- pCambia-Rev	cagttggaattctagaTCAGTTTGCTGGAAAACTCGA
SlWRKY22- pCambia-For	ttacgaatttcgacctgcagATGGAAGAGGATTGGGATCT
SlWRKY22- pCambia-Rev	cagttggaattctaga TCAAACGCCGCCTGCGG
SlWRKY25- pCambia-For	ttacgaatttcgacctgcagATGGAAGAAGATTGGGATCTAA
SlWRKY25- pCambia-Rev	cagttggaattctagaTCAACGACCGCCTGCCGC
SlWRKY31- pCambia-For	ttacgaatttcgacctgcagATGGCTGCTTCAAGTTTCTC
SlWRKY31- pCambia-Rev	cagttggaattctagaTCAGCAAAGCAATGACTCC
SlWRKY33- pCambia-For	ttacgaatttcgacctgcagATGGCTTCTTCAGGTGGAAAT
SlWRKY33- pCambia-Rev	cagttggaattctaga TCAGTTAAGGAAAGAGCTGAAG
SlWRKY53- pCambia-For	ttacgaatttcgacctgcagATGGATTGTGCATCAAACTG
SlWRKY53- pCambia-Rev	cagttggaattctagaTCATGAGAAAAATTTGGGGTT
SlWRKY54- pCambia-For	ttacgaatttcgacctgcag ATGGATTGTGGATTCAATTATGA
SlWRKY54- pCambia-Rev	cagttggaattctaga TTATCTGAAAAAATCAGAGAAATT

Note: The letters of lowercases are linearized homologous sequences ends of *pCambia35tlegyfps* vectors.

Table S9. The primers used for construction pGBKT7-DBD-WRKYx vector

Name of Genes	Primers（5′-3′）	Enzyme sites
SlWRKY16-DBD-For	CATGccatggAGATGGATAAAGGATGGGGACTT	*Nco* I
SlWRKY16-DBD-Rev	ACGCgtcgacGCTACTGTTCTTTTCCAGGACCA	*Sal* I
SlWRKY17-DBD-For	CGCggatccGTATGGCCAAAGGAAGTGGACTCT	*BamH* I
SlWRKY17-DBD-Rev	ACGCgtcgacGTCAGTTTGCTGGAAAACTCGAAT	*Sal* I
SlRIN-DBD-For	tcagaggaggacctgcatatgATGGGTAGAGGGAAAGTAGAAT	Note
SlRIN-DBD-Rev	tcgacggatccccgggaattcAAGCATCCATCCAGGTACA	Note
SlERF7-DBD-For	tcagaggaggacctgcatatgATGGCTAGGGCACAACAA	Note
SlERF7-DBD-Rev	tcgacggatccccgggaattcTCACTGGATTTGGTGAGAAGG	Note
SlERF2b-DBD-For	tcagaggaggacctgcatatgATGTGTGGTGGTGCAATTC	Note
SlERF2b-DBD-Rev	tcgacggatccccgggaattcTTAAACTACATTATAACTTGGTTGT	Note

Note: The letters of lowercases are linearized homologous sequences ends of BD vector

Table S10. The primers used for construction BiFC vector

Name of Genes	Primers（5′ -3′ ）
SlWRKY16- BiFC-For	tcagatctcgagctcaagcttATGGATAAAGGATGGGGACT
SlWRKY16-BiFC-Rev	gactctagatcaggtggatccCTGTTCTTTTCCAGGACC
SlWRKY17-BiFC-For	tcagatctcgagctcaagcttATGGCCAAAGGAAGTGGA
SlWRKY17-BiFC-Rev	gactctagatcaggtggatccGTTTGCTGGAAAACTCGAA
SlWRKY 33-BiFC-For	tcagatctcgagctcaagcttATGGCTTCTTCAGGTGGAAAT
SlWRKY 33-BiFC-Rev	gactctagatcaggtggatccGTTAAGGAAAGAGCTGAAGAAT
SlWRKY 53-BiFC-For	tcagatctcgagctcaagcttATGGATTGTGCATCAAACTGG
SlWRKY 53-BiFC-Rev	gactctagatcaggtggatccTGAGAAAAATTTGGGGTTACCAA
SlWRKY 54-BiFC-For	tcagatctcgagctcaagcttATGGATTGTGGATTCAATTATGAAT
SlWRKY 54-BiFC-Rev	gactctagatcaggtggatccTCTGAAAAAATCAGAGAAATTTGG
SlERF2b-BiFC-For	tcagatctcgagctcaagcttATGTGTGGTGGTGCAATTCT
SlERF2-BiFC-Rev	gactctagatcaggtggatccAACTACATTATAACTTGGTTGTAC
SlERF7-BiFC-For	tcagatctcgagctcaagcttATGGCTAGGGCACAACAA
SlERF7-BiFC-Rev	gactctagatcaggtggatccCTGGATTTGGTGAGAAGGAA
SlRIN-BiFC-For	tcagatctcgagctcaagcttATGGGTAGAGGGAAAGTAGAA
SlRIN-BiFC-Rev	gactctagatcaggtggatccAAGCATCCATCCAGGTACA

Table S11. The primers used for construction VIGS vector

Gene name	Primer (5'- 3')
LIC-*SlWRKY16*-For	CGACGACAAGACCCTGGGTTTAATCATATGATGAACCG
LIC-*SlWRKY16*-Rev	GAGGAGAAGAGCCCTAATTGGAATTATGAGATAGATCGT
LIC -*SlWRKY17*-For	CGACGACAAGACCCTCATAACAACCAATTTTATCCTAAGA
LIC-*SlWRKY17*-Rev	GAGGAGAAGAGCCCTAACATTGAATCTAGTTCCACAT
LIC -*SlWRKY22*-For	CGACGACAAGACCCTCATGCGGTGGTCAGAGGC
LIC-*SlWRKY22*-Rev	GAGGAGAAGAGCCCTGGTCTTGGGCTTTGAACATGTG
LIC -*SlWRKY23*-For	CGACGACAAGACCCTATTTCATTGACAACAACGGTT
LIC-*SlWRKY25*-Rev	GAGGAGAAGAGCCCTTTTGATTGTTTGACTTTGAGCA
LIC -*SlWRKY31*-For	CGACGACAAGACCCTTGACGACTTCTTTCACCGACCTT
LIC-*SlWRKY31*-Rev	GAGGAGAAGAGCCCTTGTGGGCTCTTGACAATTCCA
LIC -*SlWRKY33*-For	CGACGACAAGACCCTAACTCATTTTCTTCTTCTCAATTC
LIC-*SlWRKY33*-Rev	GAGGAGAAGAGCCCTTTCACTCCAGTTTTTGCTG
LIC -*SlWRKY53*-For	CGACGACAAGACCCTTATTCTGCACCACAACCAAC
LIC-*SlWRKY53*-Rev	GAGGAGAAGAGCCCTTTGGATCCAGCTCCAAATG
LIC -*SlWRKY54*-For	CGACGACAAGACCCTTGCTGCTGCTACACAACCA
LIC-*SlWRKY54*-Rev	GAGGAGAAGAGCCCTCTACATGTTCAAGTGGAAACTC
LIC -*SlACS4*-For	CGACGACAAGACCCTTCTCCACTTTCACTAACG
LIC -*SlACS4*-Rev	GAGGAGAAGAGCCCTGCAAATCCATCCAACAATA

Note： The nucleotides underlined are LIC sequences.

References

1. Giovannoni, J. J. *et al.* Molecular genetic analysis of theripening-inhibitor andnon-ripening loci of tomato: A first step in genetic map-based cloning of fruit ripening genes. *Mol. Gen. Genet.* **248**, 195-206 (1995).
2. Martel, C., Vrebalov, J., Tafelmeyer, P. & Giovannoni, J. J. The tomato MADS-box transcription factor RIPENING INHIBITOR interacts with promoters involved in numerous ripening processes in a COLORLESS NONRIPENING-dependent manner. *Plant Physiol.* **157**, 1568-1579 (2011).
3. Li, J.-b. & Luan, Y.-s. Molecular cloning and characterization of a pathogen-induced WRKY transcription factor gene from late blight resistant tomato varieties Solanum pimpinellifolium L3708. *Physiol. Mol. Plant P.* **87**, 25-31 (2014).
4. Liu, B. *et al.* Tomato WRKY transcriptional factor SlDRW1 is required for disease resistance against Botrytis cinerea and tolerance to oxidative stress. *Plant Sci.* **227**, 145-156 (2014).
5. Molan, Y. & El-Komy, M. Expression of Sl-WRKY1 transcription factor during B. cinerea tomato interaction in resistant and susceptible cultivars. *International Journal of Plant Breeding and Genetics* **4**, 1-12 (2010).
6. Bhattarai, K. K., Atamian, H. S., Kaloshian, I. & Eulgem, T. WRKY72-type transcription factors contribute to basal immunity in tomato and Arabidopsis as well as gene-for-gene resistance mediated by the tomato R gene Mi-1. *Plant J* **63**, 229-240 (2010).
7. Atamian, H. S., Eulgem, T. & Kaloshian, I. SlWRKY70 is required for Mi-1-mediated resistance to aphids and nematodes in tomato. *Planta* **235**, 299-309 (2012).
8. Huang, S. *et al.* Genome-wide analysis of WRKY transcription factors in Solanum lycopersicum. *Mol. Genet. Genomics* **287**, 495-513 (2012).
9. Consortium, T. G. The tomato genome sequence provides insights into fleshy fruit evolution. *Nature* **485**, 635-641 (2012).
10. Robatzek, S. & Somssich, I. E. A new member of the Arabidopsis WRKY transcription factor family, AtWRKY6, is associated with both senescence ‐ and defence ‐ related processes. *Plant J* **28**, 123-133 (2001).
11. Robatzek, S. & Somssich, I. E. Targets of AtWRKY6 regulation during plant senescence and pathogen defense. *Genes Dev.* **16**, 1139-1149 (2002).
12. Chen, L., Zhang, L. & Yu, D. Wounding-induced WRKY8 is involved in basal defense in Arabidopsis. *Mol. Plant-Microbe In.* **23**, 558-565 (2010).
13. Potschin, M., Schlienger, S., Bieker, S. & Zentgraf, U. Senescence networking: WRKY18 is an upstream regulator, a downstream target gene, and a protein interaction partner of WRKY53. *J Plant Growth Regul.* **33**, 106-118 (2014).
14. Zhou, X., Jiang, Y. & Yu, D. WRKY22 transcription factor mediates dark-induced leaf senescence in Arabidopsis. *Mol. Cells* **31**, 303-313 (2011).
15. Besseau, S., Li, J. & Palva, E. T. WRKY54 and WRKY70 co-operate as negative regulators of leaf senescence in Arabidopsis thaliana. *J Exp Bot* **63**, 2667-2679, doi:10.1093/jxb/err450 (2012).
16. Li, G. *et al.* Dual-level regulation of ACC synthase activity by MPK3/MPK6 cascade and its downstream WRKY transcription factor during ethylene induction in Arabidopsis. *PLoS Genet.* **8** (2012).
17. Hinderhofer, K. & Zentgraf, U. Identification of a transcription factor specifically expressed at the onset of leaf senescence. *Planta* **213**, 469-473 (2001).

18. Miao, Y., Laun, T., Zimmermann, P. & Zentgraf, U. Targets of the WRKY53 transcription factor and its role during leaf senescence in Arabidopsis. *Plant Mol. Biol.* **55**, 853-867 (2004).
19. Miao, Y., Smykowski, A. & Zentgraf, U. A novel upstream regulator of WRKY53 transcription during leaf senescence in Arabidopsis thaliana. *Plant Biol.* **10**, 110-120 (2008).
20. Jiang, Y., Liang, G., Yang, S. & Yu, D. Arabidopsis WRKY57 functions as a node of convergence for jasmonic acid–and auxin-mediated signaling in jasmonic acid–induced leaf senescence. *Plant Cell* **26**, 230-245 (2014).
21. Ulker, B., Shahid Mukhtar, M. & Somssich, I. E. The WRKY70 transcription factor of Arabidopsis influences both the plant senescence and defense signaling pathways. *Planta* **226**, 125-137 (2007).
22. Li, Z., Peng, J., Wen, X. & Guo, H. Gene network analysis and functional studies of senescence-associated genes reveal novel regulators of Arabidopsis leaf senescence. *J Integr. Plant Biol.* **54**, 526-539 (2012).
23. Jing, S., Zhou, X., Song, Y. & Yu, D. Heterologous expression of OsWRKY23 gene enhances pathogen defense and dark-induced leaf senescence in Arabidopsis. *Plant Growth Regul.* **58**, 181-190 (2009).
24. Liu, X., Bai, X., Wang, X. & Chu, C. OsWRKY71, a rice transcription factor, is involved in rice defense response. *J Plant Physiol.* **164**, 969-979 (2007).
25. Ricachenevsky, F. K., Sperotto, R. A., Menguer, P. K. & Fett, J. P. Identification of Fe-excess-induced genes in rice shoots reveals a WRKY transcription factor responsive to Fe, drought and senescence. *Mol. Biol. Rep.* **37**, 3735-3745 (2010).
26. Zhang, S.-L. *et al.* GbWRKY1, a novel cotton (Gossypium barbadense) WRKY gene isolated from a bacteriophage full-length cDNA library, is induced by infection with Verticillium dahliae. (2012).
27. Wang, X. *et al.* GhWRKY40, a multiple stress-responsive cotton WRKY gene, plays an important role in the wounding response and enhances susceptibility to Ralstonia solanacearum infection in transgenic Nicotiana benthamiana. *PloS one* **9**, e93577 (2014).
28. Yang, B., Jiang, Y., Rahman, M. H., Deyholos, M. K. & Kav, N. N. Identification and expression analysis of WRKY transcription factor genes in canola (Brassica napus L.) in response to fungal pathogens and hormone treatments. *BMC Plant Biol.* **9**, 68 (2009).
29. Zhang, Z., Zhang, H., Quan, R., Wang, X.-C. & Huang, R. Transcriptional regulation of the ethylene response factor LeERF2 in the expression of ethylene biosynthesis genes controls ethylene production in tomato and tobacco. *Plant Physiol.* **150**, 365-377 (2009).
30. Guo, F., Zhou, W., Zhang, J., Xu, Q. & Deng, X. Effect of the citrus lycopene β-cyclase transgene on carotenoid metabolism in transgenic tomato fruits. *PloS one* **7**, e32221 (2012).
